# Identifying Protein–metabolite Networks Associated with COPD Phenotypes

**DOI:** 10.3390/metabo10040124

**Published:** 2020-03-25

**Authors:** Emily Mastej, Lucas Gillenwater, Yonghua Zhuang, Katherine A. Pratte, Russell P. Bowler, Katerina Kechris

**Affiliations:** 1 Computational Bioscience Program, University of Colorado Anschutz Medical Campus, Aurora, CO 80045, USA; 2 National Jewish Health, Denver, CO 80206, USAprattek@njhealth.org (K.A.P.);; 3 Colorado School of Public Health, University of Colorado Anschutz Medical Campus, Aurora, CO 80045, USA; 4 School of Medicine, University of Colorado Anschutz Medical Campus, Aurora, CO 80045, USA

**Keywords:** proteomics, metabolomics, COPD, SmCCNet, multi-omic networks

## Abstract

Chronic obstructive pulmonary disease (COPD) is a disease in which airflow obstruction in the lung makes it difficult for patients to breathe. Although COPD occurs predominantly in smokers, there are still deficits in our understanding of the additional risk factors in smokers. To gain a deeper understanding of the COPD molecular signatures, we used Sparse Multiple Canonical Correlation Network (SmCCNet), a recently developed tool that uses sparse multiple canonical correlation analysis, to integrate proteomic and metabolomic data from the blood of 1008 participants of the COPDGene study to identify novel protein–metabolite networks associated with lung function and emphysema. Our aim was to integrate -omic data through SmCCNet to build interpretable networks that could assist in the discovery of novel biomarkers that may have been overlooked in alternative biomarker discovery methods. We found a protein–metabolite network consisting of 13 proteins and 7 metabolites which had a −0.34 correlation (*p*-value = 2.5 × 10^−28^) to lung function. We also found a network of 13 proteins and 10 metabolites that had a −0.27 correlation (*p*-value = 2.6 × 10^−17^) to percent emphysema. Protein–metabolite networks can provide additional information on the progression of COPD that complements single biomarker or single -omic analyses.

## 1. Introduction

Chronic obstructive pulmonary disease (COPD) is the fourth leading cause of death in the United States [1]. While COPD patients have a history of tobacco smoke exposure, only a minority of smokers develop COPD [2]. Furthermore, while COPD is strictly defined using spirometry to document airflow obstruction, there are other clinical COPD phenotypes such as chronic bronchitis (defined clinically by cough and sputum production) and emphysema (defined radiographically by loss of lung tissue with replacement by air), which may occur without airflow obstruction as well as have poor correlations to airflow obstruction [3]. Although smoke exposure drives COPD, we still have a poor understanding of the molecular phenotypes that are associated with specific phenotypes [4]. Most biomarker investigations have focused on single molecules (e.g., alpha-1 antitrypsin, sRAGE) [5,6]; however, no single molecule can fully explain the development of COPD. For example, the only well-established genetic risk (alpha-1 antitrypsin deficiency) accounts for only 1–2% of COPD cases [7]. Other studies have implicated proteases, oxidative stress, immune defects, and infections as causes of COPD [8]. While single biomarker studies may facilitate prognosis and allow for individualized treatment, panels of several biomarkers have been shown to improve predictive value compared to single biomarkers [9]. This suggests that network analysis could be utilized to explore multiple biomarkers and their combined relationship to COPD.

Networks are a natural framework to represent relationships between molecular components [10]. A network consists of a series of nodes, or biological entities such as metabolites and proteins. Connecting these nodes are edges, which represent relationships between nodes and are used to infer both indirect and direct molecular interactions, such as protein–protein interactions [11,12]. The use of networks is an important framework for understanding systems biology because they provide structure to complex data and can give us a graphical representation of molecular interactions [13].

The two most common -omics approaches to studying COPD use either lung-derived samples (tissue or bronchoalveolar lavage fluid) or blood. While lung -omics may be closer to the target organ in COPD, obtaining lung samples carries a moderate or high risk as well as is expensive, and it is unlikely that a lung sample-derived -omics signature would be used in clinical practice. In contrast, obtaining a blood sample carries a low risk and is more affordable. Furthermore, there is evidence that COPD has systemic effects such as bone loss, depression, weight loss, and hypertension, and thus it is reasonable to use blood assays for identifying biomarkers [14]. For this study, we integrated blood-based proteomic and metabolomic data to build protein–metabolite networks that were associated with COPD. A recently developed tool called Sparse Multiple Canonical Correlation Network (SmCCNet) [15] uses a canonical correlation-based approach to simultaneously integrate multi-omics data and a phenotype of interest to build interpretable networks.

Unlike pairwise correlations between individual features, canonical correlation measures the relatedness of two sets of features simultaneously by finding a linear combination of members from each set. SmCCNet is an extension of canonical correlation in which linear combinations are found to maximize the correlation between multi-omics datasets (e.g., metabolite, protein) and a phenotype of interest (e.g., emphysema). The formal and complete method of SmCCNet has been published in Shi et al. 2019 [15]. The authors also inferred mRNA–miRNA networks associated with COPD phenotypes in a small set of 27 subjects. In this study, we used SmCCNet to integrate proteomic and metabolomic data from 1008 participants of the COPDGene study, which is the most comprehensive set of blood protein and metabolite biomarker data available to date, to identify novel protein–metabolite networks associated with lung function and emphysema. Our aim was to integrate -omic data through SmCCNet to build interpretable networks that could assist in the discovery of novel biomarkers that might have been overlooked in alternative biomarker discovery methods.

## 2. Results

### 2.1. Introduction

For this study, we aimed to combine multi-omics data with clinical phenotypes to discover novel molecular connections that may otherwise go unnoticed. More specifically, we aimed to find protein–metabolite networks that were correlated with important COPD phenotypes. We applied SmCCNet to proteomic and metabolomic data while focusing on forced expiratory volume in one second (FEV_1_ percent predicted (FEV_1_%)) and percent emphysema (see Methods). In human studies, covariates may influence protein and metabolite abundance. To account for the potential influence, covariates (e.g., white blood cell count, percent eosinophil, percent lymphocytes, percent monocytes, percent neutrophils, and hemoglobin) are adjusted from the proteomic and metabolomic data. However, there is no scientific consensus on the effect covariate adjustment has on analysis because many covariates can also be associated with disease. Therefore, we present the results of SmCCNet applied to unadjusted proteomic and metabolomic data as an additional analysis in the Supplementary Materials. For each phenotype network analysis, we present the identified network as well as individual network node analysis. We then discuss the clinical relevance of network hubs, which are nodes with high connectivity (i.e., node with many edges to other nodes). Finally, we present a secondary analysis of the networks and associations with study cohort subpopulations (e.g., moderate and severe COPD, frequency of exacerbations, and heart disease comorbidities).

### 2.2. Correlations between Adjusted -Omic Data and Phenotype

Subjects profiled were recruited as part of the COPDGene study (see Methods) and are balanced with respect to control and COPD status and are predominately white and former smokers (Table 1). Data processing for the two -omic datasets is described in the Methods. Before applying SmCCNet to -omic data, we explored the range of correlations between -omic datasets and between -omic data and the phenotype of interest. The range of correlations between the adjusted proteomic data and the adjusted metabolomic data was −0.51 to 0.67, but the range of correlations between the adjusted metabolomic or adjusted proteomic data with either of the phenotypes was smaller and in the −0.21 to 0.24 range (Figure S1). With many features involved in the same pathway, it is not unexpected that the range of correlations between the adjusted proteomic and metabolomic data was larger than the range of correlations between the -omic datasets and the phenotypes. However, this discrepancy can result in networks that are driven by proteomic to metabolomic correlations and ignore potentially important correlations between -omic data and the phenotype. Therefore, additional emphasis was made on the correlations between -omic data and the phenotypes when SmCCNet was applied to the proteomic and metabolomic data (e.g., scaling constants increased; see Methods and Supplementary Materials for more detail).

### 2.3. Identified Network Associated with FEV_1_%

To identify a network associated with FEV_1_%, we focused on optimizing the scaling constants and network edge threshold. Final parameter selection was made by a variety of diagnostics including the correlation of each resulting network’s first principal component to FEV_1_%, ratio of protein to metabolite nodes, and strength of network edges. Using principal component analysis (PCA), the first PC (PC1) is a single summary of the network to help with interpretation and explains the most variance. We aimed for a network with a high PC1 correlation to FEV_1_% (|rho| ≥ 0.20). We also aimed for a network with the strongest connections (network edges with largest values); edges represent the level of association between metabolite–protein pairs relative to FEV_1_%. Lastly, we aimed to find networks that had near equal proportions of metabolites and proteins since our intent was to find protein–metabolite networks. We wanted to avoid networks that were driven by protein–protein correlations or metabolite–metabolite correlations.

The final selected scaling constant of 11 was applied to SmCCNet (Table S1, Figure S2; see Supplementary Materials for more detail). To better visualize the strongest network connections, edges with values less than 0.004 were removed (Figure S3). The final network had a −0.34 correlation to FEV_1_% (*p*-value = 2.5 × 10^−28^). As a comparison, the network that included all proteins and metabolites that could not be clustered into the identified network had a much lower correlation (rho = 0.08, *p*-value = 0.011) with FEV_1_% (see Supplementary Materials for more detail).

**Figure 1:**
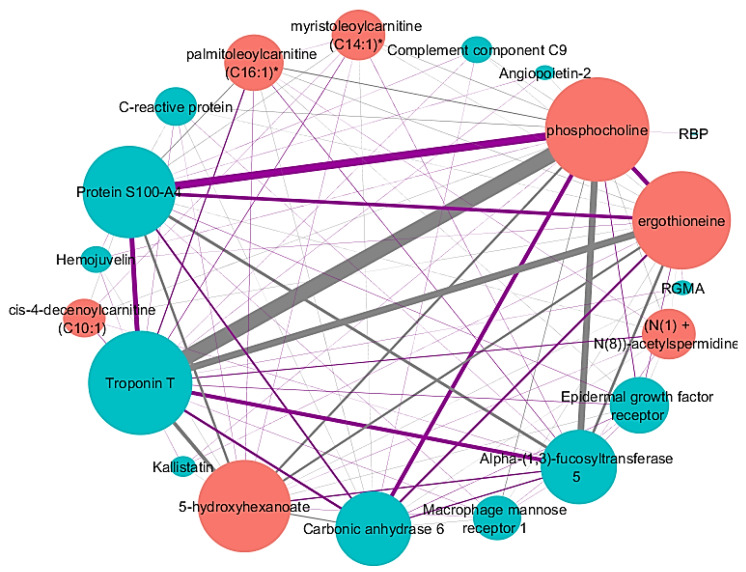
Identified network after applying sparse multiple canonical correlation network (SmCCNet) to adjusted proteomic and metabolomic data and FEV_1_%. Proteins are blue nodes and metabolites are red nodes. Grey edges represent a negative correlation between the nodes. Purple edges represent a positive correlation between the nodes. Edge thickness corresponds to the relationships between the nodes based on the canonical weights. Node size corresponds with connectivity. Abbreviations: retinol-binding protein (RBP), repulsive guidance molecule A (RGMA). * Indicates a compound that has not been confirmed based on a standard, but Metabolon is confident in its identity.

The identified network for FEV_1_% had thirteen proteins and seven metabolites with a varying range of individual feature correlations with FEV_1_% (Figure 1, Table 2). The network also displayed a high level of connectivity, with each node being connected to multiple nodes, illustrating that the proteins and metabolites have multiple relationships with each other as well as a correlation to FEV_1_%. Network hubs, nodes that have a high level of connectivity (i.e., number of edges connected to the node), included troponin T and phosphocholine with 18 edges each. The strongest edge connected troponin T and phosphocholine, demonstrating that the two features have the highest pairwise correlation to FEV_1_%. Other nodes with high connectivity include the metabolites ergothioneine and 5-hydroxyhexanoate and protein S100-A4—all of which are connected to each other by strong edges.

### 2.4. Network Comparison between Adjusted and Unadjusted -Omic Data with FEV_1_%

Proteomic and metabolomic data from human studies in blood may be influenced by covariates such as white blood cell count and percent monocytes. However, there is a lack of consensus on the best way to account for covariates within data and how much of an effect covariate adjustment has on the data. Therefore, we applied SmCCNet to adjusted (presented earlier) and unadjusted proteomic and metabolomic data in parallel to compare the results through a sensitivity analysis (Figures S4–S7, Tables S2 and S3).

When comparing the FEV_1_% protein–metabolite network constructed on the adjusted -omic data (Figure 1) with the network from the unadjusted -omic data (Figure S7), there was a significant number of protein and metabolite nodes consistent between the two networks (Fisher’s exact test *p*-value = 8.2 × 10^−20^) (Table S4). Troponin T was a node with high connectivity in the adjusted network and was the main hub in the unadjusted network. Other proteins such as epidermal growth factor receptor and protein S100-A4 were found in both networks. Metabolites that had high connectivity and strong edges in the adjusted FEV_1_% network, such as phosphocholine and ergothioneine, were also found in the unadjusted network.

While the network nodes were similar between the adjusted and unadjusted FEV_1_% networks, the number of edges and network topology were different between the two. The network constructed on adjusted -omic data was a densely connected network with a mesh-like appearance where all nodes, except RBP, have multiple connections to other proteins and metabolites. The network constructed from the unadjusted network, on the other hand, more closely resembled a star topology with troponin T at the center of the network.

### 2.5. Identified Network Associated with Percent Emphysema

Similar to methods used to construct protein–metabolite networks correlated with FEV_1_%, we optimized the scaling constant and the edge threshold to identify a network associated with percent emphysema. The final selected scaling constant of 15 was applied to SmCCNet (Table S5, Figure S8). Edges with values less than 0.5 were removed from the network to better visualize the strongest network connections (Figure S9). The final network had a −0.27 correlation to percent emphysema (*p*-value = 2.6 × 10^−17^). As a comparison, the proteins and metabolites not included in this network had a lower correlation with percent emphysema (rho = −0.076, *p*-value = 0.019; see Supplementary Materials for more detail).

**Figure 2:**
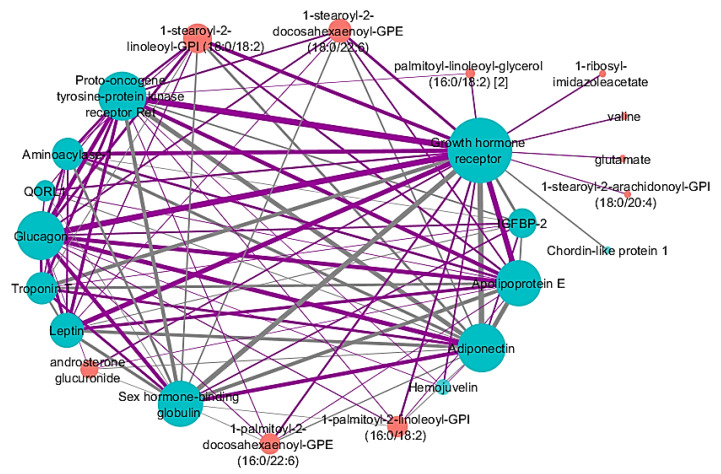
Identified network after applying SmCCNet to adjusted proteomic and metabolomic data and percent emphysema. Proteins are blue nodes and metabolites are red nodes. Grey edges represent a negative correlation between the nodes. Purple edges represent a positive correlation between the nodes. Edge thickness corresponds to the relationships between the nodes based on the canonical weights. Node size corresponds with connectivity. Abbreviations: insulin-like growth factor-binding protein 2 (IGFBP-2), quinone oxidoreductase-like protein 1 (QORL1).

The identified network associated with percent emphysema has 13 proteins and 10 metabolites, with varying range of individual feature correlations with percent emphysema (Figure 2, Table 3). Similar to the identified FEV_1_% network, the identified percent emphysema network displayed a high level of connectivity, with most nodes connected to multiple nodes. This illustrates that most of the proteins and metabolites within the network have multiple connections with other features as well as a correlation to percent emphysema. The largest network hub was growth hormone receptor, with 22 edges. Other nodes included the proteins proto-oncogene tyrosine-protein kinase receptor ret, glucagon, and adiponectin. The strongest edges in the network connected growth hormone receptor with the other hubs mentioned demonstrating that growth hormone receptor has multiple pairwise correlations to percent emphysema.

### 2.6. Network Comparison between Adjusted and Unadjusted -Omic Data with Percent Emphysema

As with FEV_1_%, SmCCNet was applied to adjusted and unadjusted proteomic and metabolomic data in parallel to compare the results for emphysema through a sensitivity analysis (Figures S10–S12, Tables S6 and S7). When comparing the percent emphysema network constructed on the adjusted -omic data (Figure 2) with the network from the unadjusted -omic data (Figure S12), there was a significant number of nodes consistent between the two networks (Fisher’s exact test *p*-value = 7.8 × 10^−20^) (Table S8). All three proteins that are in the percent emphysema unadjusted network were found in the adjusted network. Growth hormone receptor was a hub with strong edges in both networks. However, while the proteins and metabolites that made up the networks are similar, there were differences between the adjusted and unadjusted percent emphysema networks, as observed for FEV_1_%. The network constructed on adjusted -omic data was a densely connected network. Most nodes, except for five metabolites and one protein, had multiple edges connecting them to other proteins and metabolites; the other features were only connected to the growth hormone receptor. On the other hand, the network constructed from the unadjusted network more closely resembled a star topology with growth hormone receptor at the center.

### 2.7. Secondary Network Analysis

**Figure 3:**
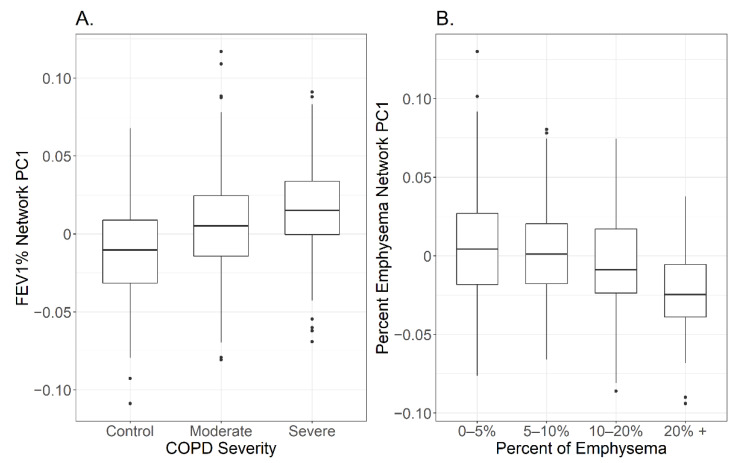
Trends of network first principal component (PC1) with disease severity. Trends within the network associated with FEV_1_% were analyzed by dividing subjects into control, moderate COPD (Gold = 1 or 2), and severe COPD (GOLD = 3 or 4) groups. There were significance differences between all three groups (*p*-value < 0.005), with higher PC1 in the severe COPD group versus the moderate COPD group versus the control (**A**). Subjects were also divided by emphysema severity to analyze trends within the network associated with percent emphysema (**B**). Only subjects that had more than 20% emphysema had a significantly different PC1 than the other three groups (*p*-value = 0.00066).

To analyze network trends between different subpopulations of COPD, changes between cohort subgroup PC1s were calculated. We first looked at trends within the network associated with FEV_1_%. A subject’s GOLD status is determined by their FEV_1_ and their FEV_1_/FVC measures. There was a −0.32 correlation between the network PC1 and FEV_1_/FVC and a −0.34 correlation between PC1 and FEV_1_% (as reported above). Subjects were also divided into a control group, a moderate COPD group (defined as GOLD = 1 or 2), and a severe COPD group (defined as GOLD = 3 or 4). There were significant differences between the three COPD group PC1s, with a significantly higher PC1 in the severe COPD group versus the moderate COPD group (*p*-value < 0.005), as well as significantly higher PC1 in the moderate COPD group versus the control group (*p*-value < 0.00001) (Figure 3A). This suggests that the associations with the FEV_1_% adjusted network are proportional to severity in airflow obstruction and not limited to severe COPD. Additionally, subjects that had one or more exacerbations had significantly higher network PC1s compared to subjects who had no exacerbations (*p*-value < 0.00001) paralleling results calculated from COPD severity subgroups (Figure S13A). Lastly, subjects determined to have heart disease had significantly higher PC1s compared to subjects who did not have cardiovascular disease (Figure S14A). This result is not unexpected, since heart disease is a comorbidity associated with COPD severity. However, this could explain why troponin T was a major hub in the adjusted network associated with FEV_1_%.

Trends within the adjusted network associated with percent emphysema were also analyzed. The cohort was divided into 4 groups based on their percent emphysema: 0–5% emphysema (controls), 5%–10% emphysema (mild), 10%–20% emphysema (moderate), and >20% emphysema (severe). There was a gradual decrease in the network PC1 as percent emphysema increased within the subgroups with a significant decrease between the moderate subjects with 10%–20% emphysema compared to severe subjects with more than 20% emphysema (*p*-value = 0.00066) (Figure 3B). This suggests that the most severe emphysema cases may be driving the network structure. No significant changes in PC1 were found when the cohort was divided by exacerbations or heart disease (Figures S13B and S14B).

## 3. Discussion

To our knowledge, this is the first reported study that constructs protein–metabolite networks which are correlated with COPD phenotypes. Studies have been published that construct metabolite–protein networks to gain a deeper understanding of chemical communication or to identify metabolite–protein networks important for non-COPD diseases. Piazza et al. analyzed the interactions between proteins and metabolites to map the bacterial metabolite–protein interactome and make observations on binding sites and other functional events [16]. Feng et al. constructed protein–metabolite networks to identify pathways that could regulate Cushing disease, a disease classified by a malfunctioning pituitary [17]. They revealed pathways that could have functional relationships with Cushing disease and could be used for therapeutic drug targeting. Furthermore, Zhang et al. used scaled metabolite networks in addition to scaled protein networks to prioritize COPD candidate genes [18]. However, the goal of our study, was to find networks comprised of both proteins and metabolites that could give us a better understanding of COPD phenotypes—specifically, FEV_1_% and percent emphysema.

We discovered single distinct metabolite–protein networks for two well-known clinical phenotypes of COPD, FEV_1_% and percent emphysema. For instance, the FEV_1_%-associated network had features such as C-reactive protein and mannose-binding protein and complement, as well as a strong role for troponin T, suggesting a stronger association with inflammation and heart strain. Alternatively, the network associated with percent emphysema had features such as growth hormone receptor, adipokines, amino acids, and lipids, suggesting that growth and metabolism may play a more important role in the pathogenesis of COPD. The individual correlations of the network features and network correlations were all in the correlation range of previously cited work [19,20,21].

While troponin T is a hub with high connectivity and strong edges in both the networks presented, it was more prominent in the network associated with FEV_1_%. Troponin T is a protein found in cardiac muscle fibers that aid in contraction. Patients with COPD are at an increased risk of pulmonary hypertension, which puts strain on the heart and can lead to exacerbations [22]. Elevated levels of troponin T have been found in COPD patients after an exacerbation and during right ventricular dilation [23]. S100A4 protein, a protein node connected to troponin T, is a calcium-binding protein and is involved in smooth muscle cell migration. Just as increased levels of troponin T are associated with right ventricular dilation, an increase in right ventricular pressure has been reported in mice overexpressing S100A4 [24]. S100A4 also plays a role in pulmonary vascular remodeling, which is a result of pulmonary hypertension [25]. Increased levels of troponin T and C-reactive protein (also in the network) are both associated with systemic inflammation, a symptom of COPD [26]. The activation of the complement system, a part of the immune system that promotes inflammation, occurs when C-reactive protein bind to phosphocholine-containing substances [27]. Phosphocholine, another hub in the network, is involved in inflammation; it is also an intermediate for phosphatidylcholine, a compound found in surfactant. Surfactant is the fluid that reduces surface tension in alveoli [28]. A change in regulation of phosphocholine could lead to changes in alveoli or lung inflammation. The most heavily weighted edge in this network was the connection between troponin T and phosphocholine. This edge could represent the increase in inflammation in COPD subjects that have a decrease in lung function.

Other nodes found in the network correlated with FEV_1_% also play a role in healthy pulmonary functions. Ergothioniene has been reported to protect epithelial cells against oxidative stress and benefit pulmonary macrophages [29]. Carbonic anhydrase protein is a node with a high degree of connectivity connected by heavily weighted edges. Carbonic anhydrase inhibitors, such as acetazolamide, are sometimes used for COPD therapy [30]. The carbonic anhydrase inhibitor acts as a respiratory stimulant to improve oxygenation and reduce the retention of carbon dioxide [30]. The disregulated expression of the epidermal growth factor receptor, a protein involved in cell signaling pathways, has been associated with overproduction of mucous, progression or lung fibrosis, and excessive airway proliferation [31].

While FEV_1_% assesses small airway airflow, which may be more susceptible to small airway inflammation, emphysema is a measure of lung tissue loss which could be a measure of excessive destruction, failure of growth and repair, or a combination of both. Our observed metabolite–protein network associated with emphysema identified multiple biomarkers involved in growth and repair, suggesting that emphysema is associated with an impairment in lung growth or repair. The growth hormone receptor was the largest hub within this network. Growth hormone receptor plays a role in preventing muscle atrophy and promoting skeletal muscle cell and bone growth [32]. Patients with COPD often show signs of muscle wasting as well as a shift from slow to fast-twitch muscle fibers, therefore, a change in growth hormone receptor expression could be expected [33]. Additionally, mouse models with growth hormone receptor knock-out show similar phenotypes to COPD patients such as impaired glucose tolerance [34], decreased heart function [35], and reduced muscle mass [36]. Growth hormone receptor expression can also decline as people age [37,38], especially after age 60. The median age of this study’s cohort was 68 years; therefore, growth hormone receptor could have changes in expression as a result of the cohort’s older population. Valine is a metabolite that also promotes muscle growth and overall muscle health. Jonker et al. reported that an essential amino acid supplement that includes valine, could be used as a treatment for COPD to prevent muscle wasting [39].

Leptin and adiponectin, nodes in the percent emphysema network with strong edges connecting them to growth hormone receptor, are both proteins released from adipose tissue. Adiponectin and leptin are associated with COPD, decline in lung function, and obstruction in peripheral airways [40,41]. Adiponectin-deficient mice have been reported to be protected from tobacco-induced emphysema [42], while low levels of leptin are associated with loss of respiratory muscle function and a decline in FEV_1_ [40,43]. The inverse relationship lectin and adiponectin have on COPD severity is also reflected in the protein–metabolite network correlated with percent emphysema. There is a negative edge connecting leptin and adiponectin. Apolipoprotein E, a protein expressed by alveolar macrophages and pulmonary artery smooth muscles [44], is a node that has multiple heavily weight edges connected to it.

Because covariates such as white blood cell count may influence proteomic and metabolomic data in blood from human studies, we constructed networks from both unadjusted -omic data in parallel with -omic data adjusted for cell count covariates. While the network nodes between the adjusted and unadjusted networks were similar, there were differences in the network topology. Both identified, trimmed networks constructed from adjusted data had a large number of edges with the FEV_1_% network containing 96 edges and the percent emphysema network containing 95 edges. The unadjusted networks had a smaller number of edges, with the FEV_1_% network containing 32 edges and the percent emphysema network containing 24 edges. The difference in the amount of edges also coincides with the connectivity and topology of the networks. Both adjusted networks closely resemble a mesh-like topology, with most nodes being connected to multiple proteins and networks. Alternatively, both unadjusted networks closely resemble a star topology, with one node the center of the network and all other proteins and metabolites connected to the center. Based on these results, when the covariates are not regressed out of the -omic data, relationships between proteins and metabolites could be too weak to identify. The effect the covariates have on protein and metabolite levels may be overpowering the relationship amongst proteins and metabolites. In the adjusted data, the covariates were regressed out of the data and the relationships between the proteins and metabolites appear to be stronger.

SmCCNet was initially developed to construct miRNA and mRNA networks. However, we have shown that sparse multiple canonical correlation analysis can be used on different types of -omic data including proteomic and metabolomic data. Additionally, it is beneficial to use SmCCNet for multi-omic biomarker analysis in addition to single biomarker analysis because SmCCNet allows for the discovery of biomarkers that may have otherwise been overlooked in single -omic analysis. Final networks can contain biomarkers that do not have the most significant correlations to the phenotype (|rho| < 0.15, *p*-value > 0.001) since SmCCNet considers relationships between biomarkers in addition to identifying biomarkers most highly correlated to the phenotype. Conversely, biomarkers that have a significant correlation to the phenotype (|rho| > 0.15, *p*-value < 0.001) are not guaranteed to be included in final networks if they are not highly connected to other biomarkers. While there is overlap of biomarkers discovered by both methods, it is beneficial to use SmCCNet as well as single biomarker analysis to maximize biomarker discovery. For example, in the network identified for percent emphysema, apolipoprotein E, discussed above, and IGFBP-2, a protein involved in the regulation of insulin-like growth factors [45], were both included in the trimmed network. Both proteins have relatively smaller correlations to percent emphysema, −0.13 and 0.11, respectively, compared to other proteins and metabolites that were included in the trimmed network, but are strongly connected to biomarkers that have larger correlations to percent emphysema. The inclusion of apolipoprotein E, IGFBP-2, and other metabolites and proteins that may not have the highest phenotype associations but are highly connected could lead to novel targets for intervention that otherwise might not have been detected with single -omic analysis.

A limitation to this study was that 392 unannotated metabolites were included in the dataset. This is a common limitation in studies that analyze metabolome data and is an active area of research. The annotation issue did not affect our networks that were constructed on adjusted metabolic data; however, it did affect the networks constructed on unadjusted metabolomic data. Both the network correlated to FEV_1_% (Figure S7) and percent emphysema (Figure S12) contain unannotated metabolites. Another limitation to this study was manual hyperparameter optimization. While protein–metabolite networks were constructed by selecting hyperparameters in a systematic manner, it was computationally intensive and, unfortunately, not an exhaustive search.

In conclusion, this study demonstrates that we can use sparse multiple canonical correlation analysis to integrate metabolomic and proteomic data with a phenotype of interest to build protein–metabolite networks. Because multiple proteins and metabolites may be collinear due to the strong influence of covariates such as smoking, age, and sex, it is not unexpected that the correlation between the proteomic and metabolomic data is higher than the correlation between the -omic datasets and the phenotype. Therefore, more emphasis on the -omic data to phenotype correlations may be needed when using SmCCNet to construct multi-omic networks.

While our identified networks have similarities, there were still different features and network structures which may reflect different underlying pathophysiologies between lung function and emphysema. Our identified networks could be used to identify potential proteins and metabolites that may otherwise not been considered in single biomarker or single -omic analyses.

## 4. Materials and Methods

### 4.1. COPDGene

The COPDGene study is a multicenter study that enrolled 10,198 participants with and without COPD between 2007 and 2011 (Visit 1). Five-year follow up visits took place from 2013 to 2017 (Visit 2). Study participants provided consent, and blood samples were obtained from the participants for -omics analysis [46]. In total, 1136 subjects (1040 non-Hispanic white, 96 African American) participated in an ancillary study in which they provide fresh frozen plasma collected using an 8.5 mL p100 tube (Becton Dickson) at Visit 2. After removing never smoker controls and subjects who had lung transplants, 1008 subjects had both proteomic and metabolomic profiling at Visit 2 and were used for network analysis.

### 4.2. Clinical Variables and Definitions

The following COPD phenotypes were used as clinical variables in SmCCNet: percent emphysema and percent predicted forced expiratory volume in one second (FEV_1_%). Emphysema, or the destruction of distal airspaces, is associated with the clinical severity of COPD [47] but is only loosely correlated with FEV_1_%. Percent emphysema is an imaging phenotype defined as percent of lung voxels less than −950 Hounsfield Units on inspiratory CT scans. FEV_1_% is the amount of air one can forcibly exhale in one second (L) divided by the predicted FEV_1_ adjusted for age, height, race, and sex [48]. The Global Obstructive Lung Disease (GOLD) system was used to grade COPD: GOLD 0 represents an individual without COPD (FEV_1_ ≥ 80%; FEV_1_/FVC ≥0.7), GOLD 1 (FEV_1_ ≥ 80%; FEV_1_/FVC < 0.7), GOLD 2 (50% ≤ FEV_1_ < 80%; FEV_1_/FVC < 0.7), GOLD 3 (30% ≤ FEV_1_ < 50%; FEV_1_/FVC < 0.7), and GOLD 4 (FEV_1_ < 30%; FEV_1_/FVC < 0.7), respectively represent the early, moderate, severe, and very severe stages of COPD. Preserved Ratio Impaired Spirometry (PRISm) defines individuals with a reduced FEV_1_ but with a preserved FEV_1_/FVC, where FVC is forced vital capacity (FEV_1_ < 80%; FEV_1_/FVC ≥ 0.7). FEV_1_% and percent emphysema variables were both centered and scaled. A subject was considered to have heart disease if they had at least one of the self-reported variables: atrial fibrillation, congestive heart failure, coronary artery disease, heart attack, angioplasty, coronary artery bypass graph, or coronary artery calcium greater than 100 Agatston score.

### 4.3. Proteomics and Data Processing

Proteomic data was quantified using SOMAscan^®^ Human Plasma 1.3K assay (SomaLogic, Boulder, Colorado, CO, USA) on P100 plasma at National Jewish Health. SOMAScan is a multiplex proteomic assay quantified by microarrays. This assay measured 1317 SOMAmers. SOMAmers are short single-stranded deoxyoligonucleotides (aptamers) that bind with high affinity and specificity to specific protein structures [49]. SomaLogic conducted quality assurance on each sample and normalized (hybridization and median), SOMAmers were calibrated (to remove inter-assay variation by analyte), and plate scaled (to adjust for total signal difference from plate to plate variation). As a final step, proteomic data was natural log transformed and standardized.

### 4.4. Metabolomics and Data Processing

The same P100 plasma was profiled using the Metabolon (Durham, NC, USA) Global Metabolomics platform. Briefly, untargeted gas chromatography–mass spectrometry and liquid chromatography–mass spectrometry (GC–MS and LC–MS) were used to quantify 1392 metabolites (see Supplementary Materials for more detail). A data normalization step was performed to correct variation resulting from instrument inter-day tuning differences: metabolite intensities were divided by the metabolite run day median, then multiplied by the overall metabolite median. It was determined that no further normalization was necessary based on the reduction in the significance of association between the top PCs and sample run day after normalization. Subjects with aggregate metabolite median *z*-scores greater than 3.5 standard deviation from the mean (*n* = 6) of the cohort were removed. Metabolites were excluded if >20% of samples were missing values [50]. For the 995 remaining metabolites, missing values were imputed across metabolites with k-nearest neighbors imputation (*k* = 10) using the R package ‘impute’ [51]. As a final step, metabolomic data was natural log transformed and standardized.

### 4.5. Adjusted Proteomic and Metabolomic Data

The proteomic and metabolomic data was adjusted for white blood cell count, percent eosinophil, percent lymphocytes, percent monocytes, percent neutrophils, and hemoglobin. This was performed using linear regression for each metabolite, with blood cell counts as the predictors. Residuals from these models were utilized in adjusted models moving forward. Results of running SmCCNet on unadjusted data can be found in the Supplementary Materials.

### 4.6. Statistical Package

All analyses, including SmCCNet version 0.99.0, correlations, and network sensitivity analysis, were performed with the statistical software package R v3.5.3 available on CRAN.

### 4.7. SmCCNet

Protein–metabolite networks correlated to FEV_1_% and percent emphysema were constructed using SmCCNet (Figure S15), a technique by Shi et al. [15] that uses multiple canonical correlation network analysis to integrate multi-omics data types with a phenotype of interest. The original application of SmCCNet focused on miRNA–mRNA networks. We extended SmCCNet to construct protein–metabolite networks with more rigorous hyperparameter decision making.

Before applying SmCCNet, the Pearson correlation matrices were calculated between the -omics data and the phenotype of interest. When the range of correlations between the -omic data exceeds the range of correlations between the -omic and the phenotype of interest, scaling constants are increased to prioritize the correlations between the -omic data and the phenotype of interest. Scaling constants were systematically increased to determine which value yielded the best network results. We initially applied scaling constant values of 5, 10, 15, and 20 as a first pass to decrease computational time. After reviewing network diagnostics, we further analyze scaling constant values between 2 and 20 as needed to determine the scaling constant for which the network results ceased to have a substantial change.

Since all metabolites and proteins will not contribute to the overall correlation, sparsity is imposed on the canonical correlation of SmCCNet. The sparse penalty parameters were chosen through a 5-fold cross validation (Figure S15, Step 1) to find the penalty pair that minimized prediction error. All penalty pairs from the set (0.05, 0.15, 0.25, 0.35, 0.45, 0.55) were tested in a grid search to find the optimal pair.

Lastly, after protein–metabolite networks were generated from SmCCNet, absolute edge thresholds were applied to the networks to filter out weak edges (edges with low values) [15]. Edge thresholds were systematically changed from 0 to 0.7, in increments of 0.05 to reveal trimmed, interpretable networks with strong edges that still had strong correlations to the phenotype of interest and a balanced protein to metabolite ratio.

### 4.8. Manual Hyperparameter Optimization Process

Manual hyperparameter optimization was performed to select scaling constant and edge threshold values. This process was carried out in a systematic way while taking into consideration the following results: correlation of the network to the phenotype of interest, total number of network nodes, ratio of protein to metabolite nodes, strength of network edges, and results of adjacent hyperparameters. We aimed to construct networks that had at least a 0.20 correlation to the phenotype of interest and strong edges. Edges represent pairwise correlations to the phenotype of interest. High edge values represent a high level of association between the metabolite/protein pair relative to the phenotype of interest. Lastly, we aimed to choose hyperparameters that resulted in networks that had near equal proportions of metabolites and proteins since our intent was to find protein–metabolite networks. We wanted to avoid networks that were driven by protein–protein correlations or metabolite–metabolite correlations.

### 4.9. Final Protein–Metabolite Network Correlations

To determine the strength of each network, using principal component analysis (PCA), the first PC (PC1) of the network was correlated with the phenotype of interest. PC1 was selected as the single summary of the network, since it explains the most variance in the network and helps with network interpretation. The Pearson correlation between each network node and the phenotype of interest was also calculated. Identified FEV_1_% and percent emphysema networks were visualized using Cytoscape version 3.7.2 [52].

### 4.10. Network Sensitivity Analysis

Because covariates may influence protein and metabolite abundance in human blood studies, covariates were adjusted from the proteomic and metabolomic data. White blood cell count, percent eosinophil, percent lymphocytes, percent monocytes, percent neutrophils, and hemoglobin were regressed out to create our “adjusted” datasets. Many covariates can also be associated with disease; therefore, there is no consensus on the effect covariate adjustment has on data. To parallel the results of applying SmCCNet on adjusted proteomic and metabolomic data, we present the results of SmCCNet applied to unadjusted proteomic and metabolomic data in the Supplementary Materials.

To determine how applying SmCCNet to unadjusted or adjusted proteomic and metabolic data changed the network outcome, networks that were constructed on adjusted and unadjusted -omic data were compared. Tables were constructed to compare the overlap of metabolites and proteins between the adjusted and unadjusted networks—specifically, the adjusted and unadjusted FEV_1_% networks and the adjusted and unadjusted percent emphysema networks. Fisher’s exact test was calculated to determine the significance of network node overlap. Visual comparison was also made to find patterns between network hubs and the most heavily weighted edges between the adjusted and unadjusted networks. Pearson correlations between individual proteins and metabolites and the phenotype were calculated and adjusted for the false discovery rate (FDR). Proteins and metabolites with correlations greater than 0.15 (FDR adjusted *p*-value < 0.001) were denoted as significant.

### 4.11. Secondary Network Analysis

To analyze whether a specific group within the cohort was driving the network connections, differences between network PC1 for multiple cohort subgroups was calculated by analysis of variance (ANOVA) followed by a Tukey’s honest significant difference test [53,54] when there were more than two groups. The cohort was divided by GOLD status, emphysema severity, heart disease comorbidity, and number of exacerbations. Boxplots were made to visually analyze trends for the adjusted networks associated with FEV¬_1_% and percent emphysema.

## 5. Conclusions

This study demonstrates that protein–metabolite networks associated with FEV_1_% and percent emphysema can be constructed using sparse multiple canonical correlation analysis. Manual hyperparameter optimization can be performed to select the best scaling constant and edge threshold values to yield networks that are correlated to the phenotypes of interest. Regardless of whether covariate adjustment was performed, troponin T and phosphocholine were important hubs in the network associated with FEV_1_%. Troponin T and phosphocholine are involved in systemic inflammation and may be integral in the decreased lung function of COPD patients. Growth hormone receptor, an important hub in the network associated with percent emphysema, plays a role in preventing muscle atrophy. Patients with COPD often show signs of muscle wasting, which could lead to a change in growth hormone receptor expression. In conclusion, SmCCNet allows for the integration of proteomic and metabolomic data with a phenotype of interest. We were able to identify novel networks and discover protein and metabolite connections that may have been overlooked in single biomarker discovery or single -omic methods.

## Figures and Tables

**Table 1:** Cohort characteristics.

Clinical Variables	Control (*n* = 426)	COPD (*n* = 478)	PRISm (*n* = 92)	Missing Spirometry (*n* = 12)	Whole Cohort (*n* = 1008)
Sex, % women	53.3	42.7	63	50	49.1
Race, % white	88.9	94.8	91.3	100	92.1
Former Smoker, %	73.7	77.6	70.7	66.7	75.2
Age (yr)	64.6 (58.3–71.5)	71.1 (64.9–76.6)	67.3 (60.9–72.9)	72 (68.2–75.3)	68 (61–74.6)
Body mass index (kg/m^2^)	28.4 (25.3–32.1)	27.4 (23.7–31.7)	30.8 (27.4–37.4)	27.8 (25.2–30.2)	28.1 (24.7–32.2)
Heart disease comorbidity (%)	30.3	50.2	38	50	40.7
FEV_1_% predicted	106.8 (11.7)	58(23.5)	70 (7.7)	NA	77.4 (26.6) *
Percent emphysema (% LAA < −950 HU)	2.2 (2.6)	12.8 (12.3)	1.5 (2.6)	9.2 (11.7)	7.1 (10.1) **

Data is presented as the median (interquartile range) for age and body mass index and mean (standard deviation) for FEV_1_% predicted and percent emphysema. The whole cohort is the combination of former and current smokers. PRISm: Preserved Ratio Impaired Spirometry defines individuals with a reduced FEV_1_ but with a preserved FEV_1_/FVC where FVC is forced vital capacity. GOLD: the global Obstructive Lung Disease system for grading COPD severity: GOLD 1 is early COPD, GOLD 2 is moderate COPD, GOLD 3 is severe COPD, GOLD 4 is very severe COPD, and GOLD 0 is an individual without COPD. Heart disease definition can be found in the Methods section. FEV_1_%: percent predicted forced expiratory volume in one second. Percent emphysema: percent of lung voxels less than −950 Hounsfield Units on inspiratory CT scans. * In total, 12 subjects were removed because they did not have FEV_1_ values. ** In total, 60 subjects were removed because they did not have percent emphysema values (12 control, 44 COPD, and 4 PRISm).

**Table 2:** Individual network node correlations to FEV_1_%.

-Omics Type	Network Node	Correlation to FEV_1_ (%)
**Proteins**	Troponin T	−0.254
	Protein S100-A4	0.187
	Alpha-(1,3)-fucosyltransferase 5	−0.175
	Carbonic anhydrase 6	0.171
	RGMA	0.152
	Epidermal growth factor receptor	0.151
	Hemojuvelin	0.146
	C-reactive protein	−0.144
	Macrophage mannose receptor 1	−0.144
	Kallistatin	0.141
	Angiopoietin-2	−0.140
	RBP	0.138
	Complement component C9	−0.135
**Metabolites**	Phosphocholine	0.250
	Ergothioneine	0.220
	5-hydroxyhexanoate	−0.213
	Palmitoleoylcarnitine (C16:1)	−0.205
	Myristoleoylcarnitine (C14:1)	−0.200
	Cis-4-decenoylcarnitine (C10:1)	−0.199
	(N(1) + N(8))-acetylspermidine	−0.184

Pearson correlations between FEV_1_% and individual metabolites and proteins in the identified network associated with FEV_1_%.

**Table 3:** Individual network node correlations to percent emphysema.

-Omics Type	Network Node	Correlation to Percent Emphysema
Proteins	Troponin T	0.197
	Leptin	−0.169
	Glucagon	−0.163
	Growth hormone receptor	−0.161
	Proto-oncogene tyrosine-protein kinase receptor Ret	−0.146
	Chordin-like protein 1	0.143
	Hemojuvelin	−0.142
	Sex hormone-binding globulin	0.139
	Aminoacylase-1	−0.138
	Adiponectin	0.137
	Apolipoprotein E	−0.130
	IGFBP-2	0.119
	QORL1	−0.106
Metabolites	1-stearoyl-2-linoleoyl-GPI (18:0/18:2)	−0.209
	androsterone glucuronide	−0.206
	1-stearoyl-2-docosahexaenoyl-GPE (18:0/22:6)	−0.200
	1-palmitoyl-2-docosahexaenoyl-GPE (16:0/22:6)	−0.188
	1-palmitoyl-2-linoleoyl-GPI (16:0/18:2)	−0.187
	1-ribosyl-imidazoleacetate	−0.173
	Valine	−0.168
	palmitoyl-linoleoyl-glycerol (16:0/18:2) [2]	−0.166
	1-stearoyl-2-arachidonoyl-GPI (18:0/20:4)	−0.161
	Glutamate	−0.151

Pearson correlations between percent emphysema and individual metabolites and proteins in identified network associated with percent emphysema.

## Supplementary Materials

The following are available online at https://www.mdpi.com/2218-1989/10/4/124/s1, Figure S1: Range of Correlations between Adjusted -Omic Data and Phenotypes, Figure S2: Adjusted Module Node Correlations with FEV_1_%, Figure S3: Different Edge Thresholds for the FEV_1_% Network and Adjusted -Omic Data, Figure S4: Range of Correlations between Unadjusted -Omic Data and Phenotypes, Figure S5: Unadjusted Module Node Correlations with FEV_1_%, Figure S6: Different Edge Thresholds for the FEV_1_% Network (Unadjusted -Omic Data), Figure S7: The FEV_1_% Network with Unadjusted -Omic Data, Figure S8: AdjustedModule Nodes Correlated to Percent Emphysema, Figure S9: Different Edge Thresholds for Percent Emphysema Network and Adjusted -Omic Data; Figure S10: Unadjusted Module Node Correlations to Percent Emphysema, Figure S11: Different Edge Thresholds for Unadjusted Percent Emphysema Network, Figure S12: Percent Emphysema Network with Unadjusted -Omic Data, Figure S13: Associations of Network PC1 with Exacerbations, Figure S14: Associations of Network PC1 with Heart Disease Comorbidity, Figure S15: SmCCNet Methods Outline, Table S1: Different Scaling Constants for SmCCNet Applied to FEV_1_% and Adjusted -Omic Data, Table S2: Different Scaling Constants for SmCCNet Applied to FEV_1_% and Unadjusted -Omic Data, Table S3: Individual Network Node Correlations to FEV_1_% (Unadjusted -Omic Data), Table S4: The FEV_1_% Network Nodes using Adjusted and Unadjusted Data, Table S5: Different Scaling Constants for SmCCNet Applied to Percent Emphysema and Adjusted -Omic Data, Table S6: Different Scaling Constants for SmCCNet Applied to Percent Emphysema and Unadjusted -Omic Data, and Table S7: Individual Network Node Correlations to Percent Emphysema (Unadjusted -Omic Data), Table S8: Nodes of Percent Emphysema Networks using Adjusted and Unadjusted Data.
