# Supplementary material for: Identifying Protein–metabolite Networks Associated with COPD Phenotypes
Source: Metabolites. 2020 Mar 25;10(4):124. doi: 10.3390/metabo10040124 (PMC7241079; doi:10.3390/metabo10040124)
Supplement: Supplementary file 1 [file metabolites-10-00124-s001.pdf]

Supplement Material

S.1. Correlations between Adjusted -Omic Data and Phenotype

Before applying SmCCNet to proteomic and metabolic data, we explored the range of correlations between data sets (e.g., between proteomic and metabolomic features) and between -omic data and phenotype of interest (e.g., metabolite to FEV_1%). This initial analysis is important to identify parameter options for SmCCNet, specifically the scaling constants *a*, *b*, and *c* of SmCCNet's objective function. Increasing the value of *a* prioritizes the canonical correlation between the metabolomic data set and the proteomic data set, while increasing the value of scaling constants *b* and *c* prioritize the canonical correlation between the metabolomic data (*b*) and proteomic data (*c*) with the phenotype of interest respectively. -Omic features that only have a correlation to other -omic features and not to the phenotype could be lost from the identified network if scaling constants *b* and *c* are not increased [15]. Alternately, features that have correlations to the phenotype as well as correlations to other -omic features could be included in the identified network due to the extra prioritization an increase in scaling constants *b* and *c* puts on the canonical correlations.

The range of correlations between the adjusted proteomic data and the adjusted metabolomic data was -0.51 to 0.67, but the range of correlations between the adjusted metabolomic or adjusted proteomic data with either of the phenotypes was smaller and in the -0.21 to 0.24 range (Figure S1). Therefore, scaled SmCCNet was applied to the proteomic and metabolomic data. In particular, scaled SmCCNet occurs when the scaling constant of *a* (for the two -omics data sets) is set to 1 and the scaling constants of *b* and *c* (for the phenotype correlations) are increased (e.g., we explored values up to 20, see Methods).

A.

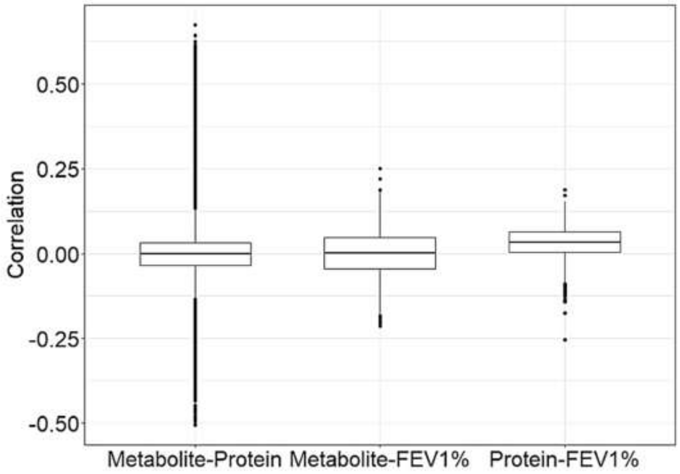

B.

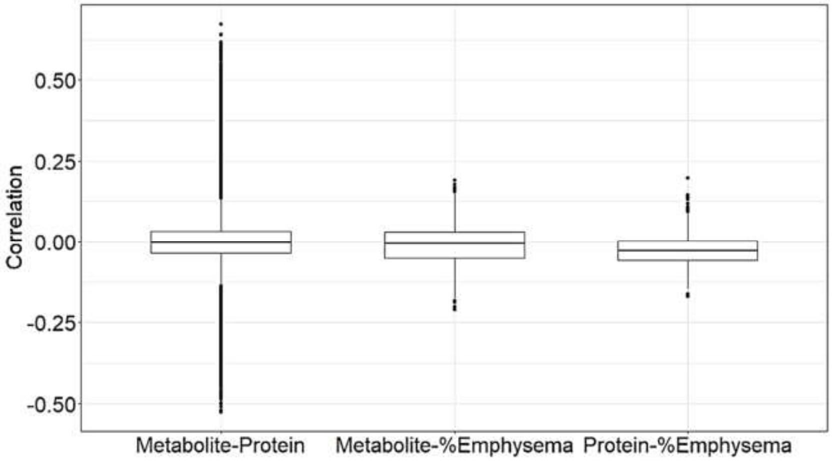

Figure S1. Range of correlations between adjusted proteomic data, adjusted metabolomic data, and FEV_1% (A) and range of correlations between adjusted proteomic data, adjusted metabolomic data, and percent emphysema (B).

S.2. Parameter Selection for FEV_1% Modules using Adjusted -Omic Datasets

We tried different scaling constants for *b* and *c* where $b = c$. After analyzing the results from each scaling constant change, a final value for the scaling constants was selected. This selection was made by a variety of diagnostics including the correlation of each resulting network module's first principal component to FEV_1%, ratio of protein to metabolite nodes, and strength of module edges. We aimed for a network with a high correlation to FEV_1% ($|rho| \geq 0.20$), close to a 1:1 ratio between proteins and metabolites, and the strongest edges amongst all derived modules. We initially applied scaling constant values of 5, 10, 15, and 20 as a first pass to decrease computational time. The diagnostics listed above indicated that we should further analyze scaling constant values between 10 and 15 to determine the scaling constant for which the network module results ceased to have a substantial change.

The different scaling constants can have a strong effect on module results and the number of modules correlated with FEV_1% (Table S1). However, after scaling constant of 12, the number of proteins, metabolites and modules do not change much. The scaling constant of 11 was selected because it results in a module that has a high correlation to FEV_1% (rho = 0.33, p-value = 3.9×10^{-26}) and a balanced ratio between proteins and metabolites in the module. Results with scaling constants below 11 have lower correlation to FEV_1% (e.g., scaling constant of 10 gives a maximum correlation between a module and FEV_1% of rho = -0.18, p-value = 2.3×10^{-8}). Because not all proteins and metabolites will contribute to the canonical correlation of SmCCNet, sparsity is imposed by adding penalties for the number of metabolites and proteins influencing the canonical weights (see Methods). The optimal metabolite and protein penalty parameters are (0.05, 0.05) when the scaling constant of 11 was applied to the SmCCNet.

Finalizing the use of scaling constant 11 (penalty parameters = (0.05, 0.05)) and applying SmCCNet to adjusted proteomic and metabolomic data and FEV_1% results in one protein-metabolite module with seven metabolites and fifteen proteins. It is important to examine not only the overall correlation of the module with FEV_1% (based on the first principal component summary), but also the individual correlations of the metabolites and proteins in the module with FEV_1% (Figure S2).

Table S1: Different Scaling Constants for SmCCNet Applied to FEV_1% and Adjusted -Omic Data

Scaling Constant (*a, b, c*)	Network Module	Correlation (p-value)	Number of Proteins	Number of Metabolites	Total Nodes
(1, 5, 5)	1	-0.12 (0.00027)	172	139	311
	2	0.16 (5.3×10^{-7})	40	21	61

	3	0.20 (3.2 × 10^{-10})	19	25	44
	4	0.14 (9.20 × 10^{-6})	98	136	234
	5	-0.13 (5.2 × 10^{-5})	7	5	12
	6	0.06 (0.053)	1	6	7
	7	0.13 (7.0 × 10^{-5})	12	6	18
	8	0.11 (0.00033)	1	2	3
	9	0.05 (0.1)	1	1	2
(1, 10, 10)	1	-0.18 (2.3 × 10^{-8})	145	351	496
	2	0.17 (6.0 × 10^{-8})	3	25	28
	3	0.08 (0.011)	1	1	2
	4	0.18 (1.9 × 10^{-8})	8	21	29
	5	0.03 (0.34)	8	1	9
(1, 11, 11)	1	0.33 (3.9 × 10^{-26})	15	7	22
(1, 12, 12)	1	0.33 (3.9 × 10^{-26})	15	7	22
(1, 13, 13)	1	0.33 (3.9 × 10^{-26})	15	7	22
(1, 15, 15)	1	0.33 (3.9 × 10^{-26})	15	7	22
(1, 20, 20)	1	-0.26 (3.7 × 10^{-17})	4	8	12

For each scaling weight change, the module diagnostics are reported. Correlation refers to the module's first principal component correlation to FEV_1%.

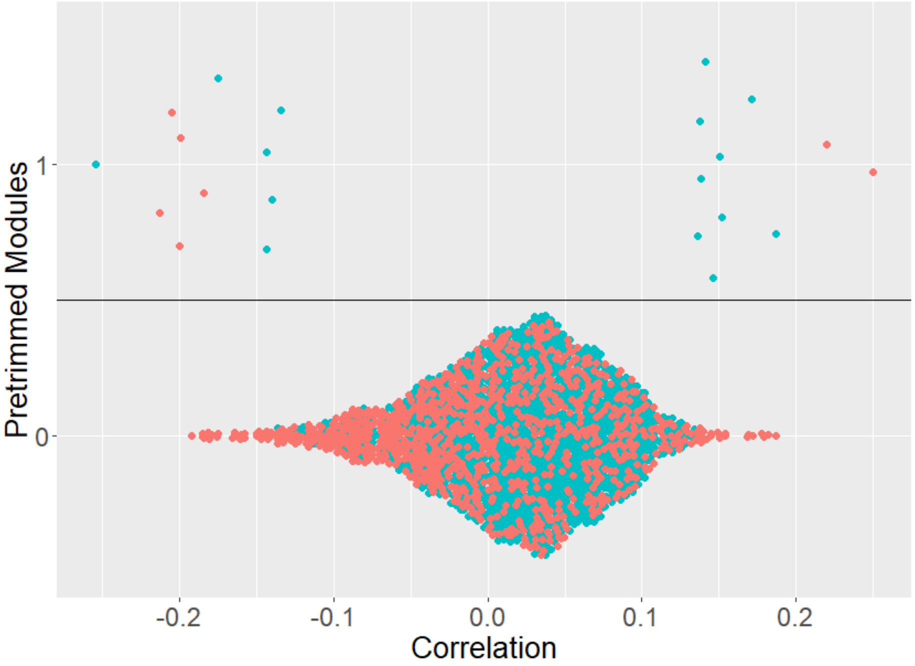

Figure S2: Module node correlations with FEV_1%. All proteins and metabolites not included in Module 1 are represented as Module 0. The proteins are blue and the metabolites are red. Module 0 has a 0.08 (p-value = 0.011) correlation with FEV_1%. Module 1 has a 0.33 (p-value = 3.9 × 10^{-26}) correlation with FEV_1%.

S.3. Identified Network Associated with FEV_1%

The initial module from SmCCNet provides a candidate network but may contain some weak edges between metabolites and proteins. By removing weaker edges, we focus on the strongest connections in the network and most informative proteins and metabolites, as they may be removed if they are weakly connected to other features. All edges are scaled based on the most heavily weighted edge on a 0 to 1 scale. The edge between troponin T and phosphocholine was so heavily weighted compared to the other edge weights, most of the edge weights were very small, or less than

0.1. Therefore, we evaluated different edge thresholds to keep the strongest connections within the network and tried values between 0 (no edges cut, keeping original module) to 0.01 in increments of 0.001. The resulting networks were summarized by the number of proteins and metabolites, in addition to the network's first principal component correlation with the phenotype (Figure S3). Edge threshold value of 0.004 was chosen because it resulted in a network with the strongest edges that still yielded the highest correlation to FEV$_1$% (rho = -0.34, p-value = 2.5 × 10^{-28}).

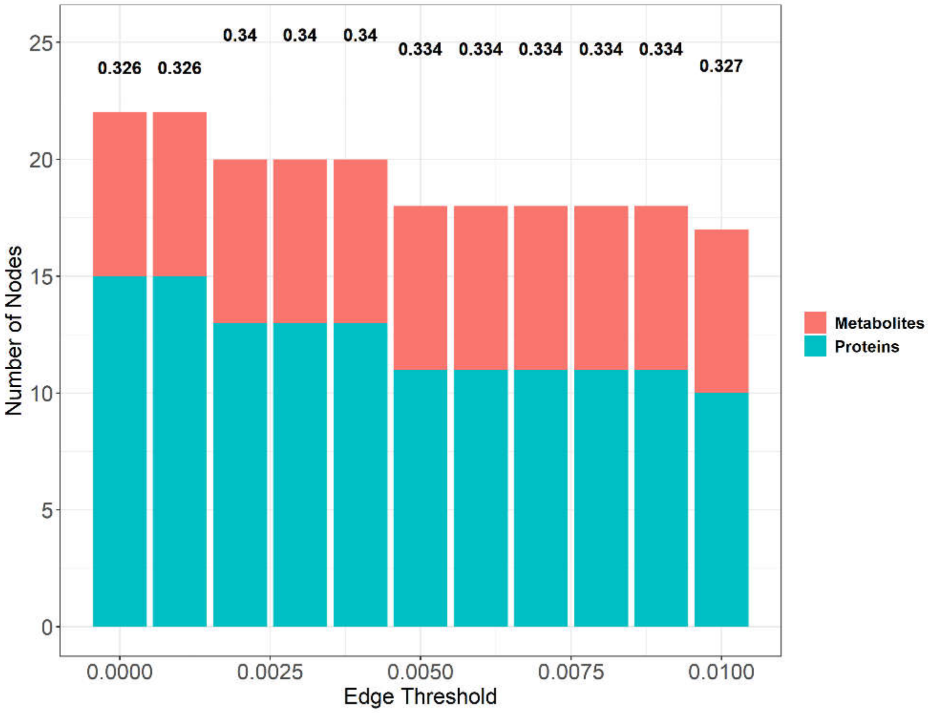

Figure S3: Effect of varying the edge threshold on the FEV$_1$% network constructed on adjusted -omic data**.** Bottom axis displays the values for edge thresholds. Number of proteins for each module are in blue and the number of metabolites for each module are in red. The correlation between the module's first principal component and FEV$_1$% is displayed as the bolded value on each bar plot. The height of the correlation corresponds to the absolute value of the correlation.

S.4. Correlations between Unadjusted -Omic Data and Phenotype of Interest

Similar to adjusted -omic data analysis, we explored the range of correlations between unadjusted -omic data sets and between -omic data sets and phenotype of interest (e.g., protein to percent emphysema) before applying SmCCNet to proteomic and metabolomic data (Figure S4). The range of correlations between the proteomic data and the metabolomic data was -0.69 to 0.74, but the range of correlations between unadjusted proteomic or metabolomic data with FEV$_1$% or percent emphysema was smaller: in the -0.31 to 0.24 range. Therefore, scaled SmCCNet was applied to the proteomic and metabolomic data.

A.

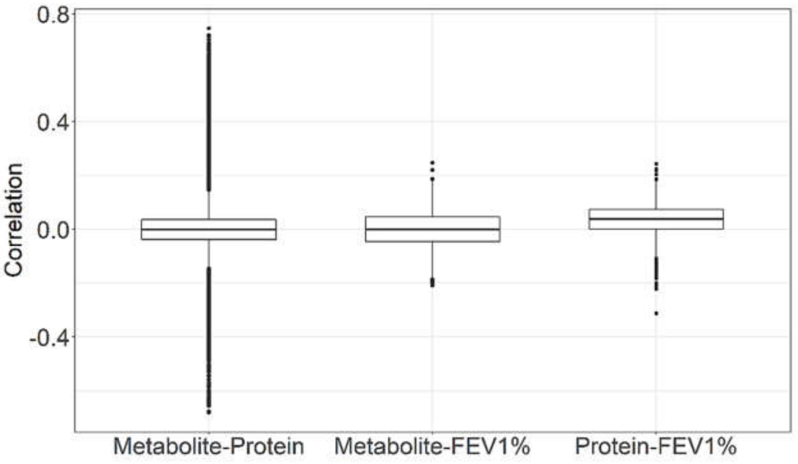

B.

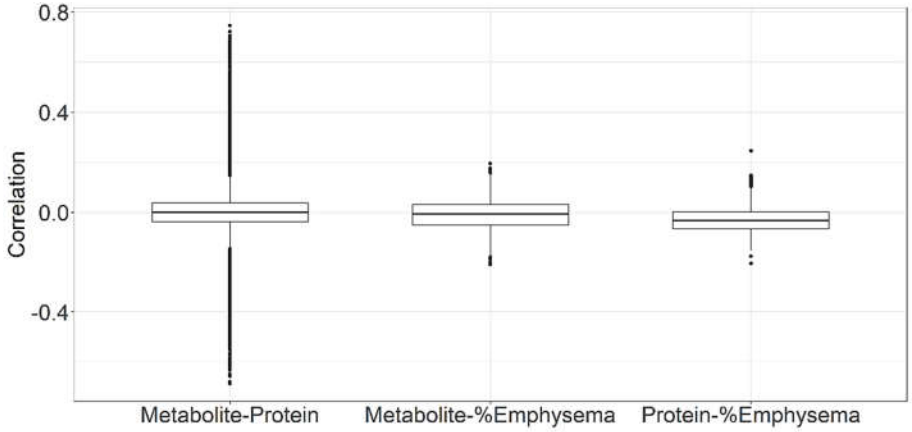

Figure S4: Range of correlations between proteomic data, metabolomic data, and FEV_1% (A) and range of correlations between proteomic data, metabolomic data, and percent emphysema (B).

S.5. FEV_1% Modules (Unadjusted -Omic Data)

We tried different scaling constants for *b* and *c* where *b* = *c* (see Methods and the main manuscript about diagnostics for selecting scaling constants*)*. Initially only scaling constant values of 11, 15, 20, and 25 were applied to SmCCNet to decrease computational time. Our diagnostics above indicated that we should further analyze scaling constants between 15 and 25.

While different scaling constant can have a strong effect on module results, after scaling constant of 19, the number of proteins, the number of metabolites, and the module correlation to FEV_1% do not change much (Table S2). Scaling constant value of 18 was selected due to module 1's high correlation to FEV_1% (rho = -0.26, p-value = 2.3×10^{-16}). This value was also the highest scaling constant value before a decrease in number of proteins in module 1. Scaling constant value of 19 resulted in a network with a higher correlation to FEV_1%, but the number of proteins in module 1 was small compared to the number of metabolites (e.g., scaling constant of 18 resulted in 131 proteins and 352 metabolites, scaling constant of 19 resulted in 9 proteins and 115 metabolites).

Finalizing the use of scaling constant 18 and applying SmCCNet to unadjusted proteomic and metabolomic data resulted in two protein-metabolite modules. The optimal penalty parameters

were (0.45, 0.25) which favors protein-metabolite networks with more metabolites and less proteins. In addition to the module's first principal component correlation to FEV_1%, individual correlations of module metabolites and proteins to FEV_1% were also reported (Figure S5). Module 0 has a much lower correlation (rho = 0.08, p-value = 0.005) with FEV_1% compared to Module 1 (rho = -0.26, p-value = 2.3×10^{-16}, 131 proteins, 352 metabolites) and Module 2 (rho = 0.16, p-value = 9.10×10^{-8}, five proteins, 10 metabolites) correlation with FEV_1%.

Table S2: Different Scaling Constants for SmCCNet Applied to FEV_1% and Unadjusted -Omic Data

Scaling Constant (*a, b, c*)	Network Module	Correlation (p-value)	Number of Proteins	Number of Metabolites	Total Nodes
(1, 11, 11)	1	-0.23 (2.1×10^{-13})	129	224	353
	2	0.25 (2.8×10^{-15})	8	8	16
	3	0.19 (1.6×10^{-9})	1	3	4
(1, 15, 15)	1	-0.23 (5.3×10^{-13})	131	363	494
	2	0.22 (2.3×10^{-12})	8	5	13
	3	0.19 (2.5×10^{-9})	31	1	32
(1, 17, 17)	1	-0.17 (1.3×10^{-7})	9	47	56
	2	-0.24 (1.5×10^{-14})	128	326	454
	3	0.19 (1.2×10^{-9})	5	8	13
	4	0.17 (6.6×10^{-8})	28	2	30
	5	-0.051 (0.11)	1	1	2
(1, 18, 18)	1	-0.26 (2.3×10^{-16})	131	352	483
	2	0.17 (9.1×10^{-8})	5	10	15
(1, 19, 19)	1	-0.31 (1.0×10^{-23})	9	115	124
(1, 20, 20)	1	-0.31 (5.8×10^{-24})	9	114	123
(1, 22, 22)	1	-0.31 (4.9×10^{-24})	9	114	123
(1, 25, 25)	1	-0.31 (4.1×10^{-24})	9	122	131

For each scaling weight change, the module diagnostics are reported. Correlation refers to the module's first principal component correlation to FEV_1%.

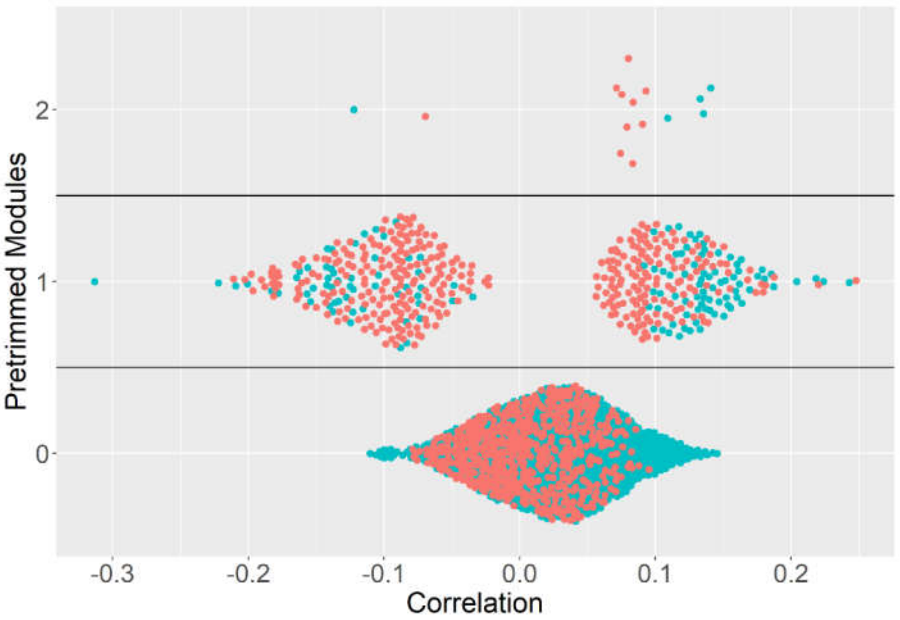

Figure S5: Module node correlations with FEV_1%. All proteins and metabolites not included in Module 1 or Module 2 are represented as Module 0. The proteins are blue and the metabolites are red. Module 0 has a 0.08 (p-value = 0.005) correlation, Module 1 has a -0.26 (p-value = 2.3×10^{-16}) correlation, and Module 2 has a 0.16 (p-value = 9.10×10^{-8}) correlation with FEV_1%.

S.6. Identified Network Associated with FEV_1% (Unadjusted -Omic Data)

The initial Module 1 provides a candidate network, but we want to focus on the proteins and metabolites connected by the strongest edges. Therefore, weaker edges and metabolites and proteins which are connected by weaker edges are removed. To keep the strongest edges, we evaluated edge threshold values between 0.4 and 0.7 at increments of 0.05. Resulting networks were summarized by network correlation to FEV_1% and number of proteins and metabolites (Figure S6). Module 1 was the only module that passed edge cuts. Edge threshold value of 0.55 was chosen as the final edge threshold because it resulted in an interpretable network with 27 nodes. It also had a high correlation (rho = -0.28, p-value = 2.7×10^{-20}) and balanced ratio of proteins to metabolites. Edge thresholds higher than 0.55 resulted in a large reduction of metabolites from the network. The identified trimmed network has 16 proteins and 11 metabolites with varying range of individual feature correlations to FEV_1% (Figure S7, Table S3). Network hubs include troponin T and epidermal growth factor receptor with the most heavily weighted edge connecting the two hubs. All other metabolites and proteins in the network are either only connected to troponin T or connected to troponin T and epidermal growth factor. Unlike the FEV_1% network created on adjusted proteomic and metabolomic data, phosphocholine is not a hub, but it is still connected to troponin T.

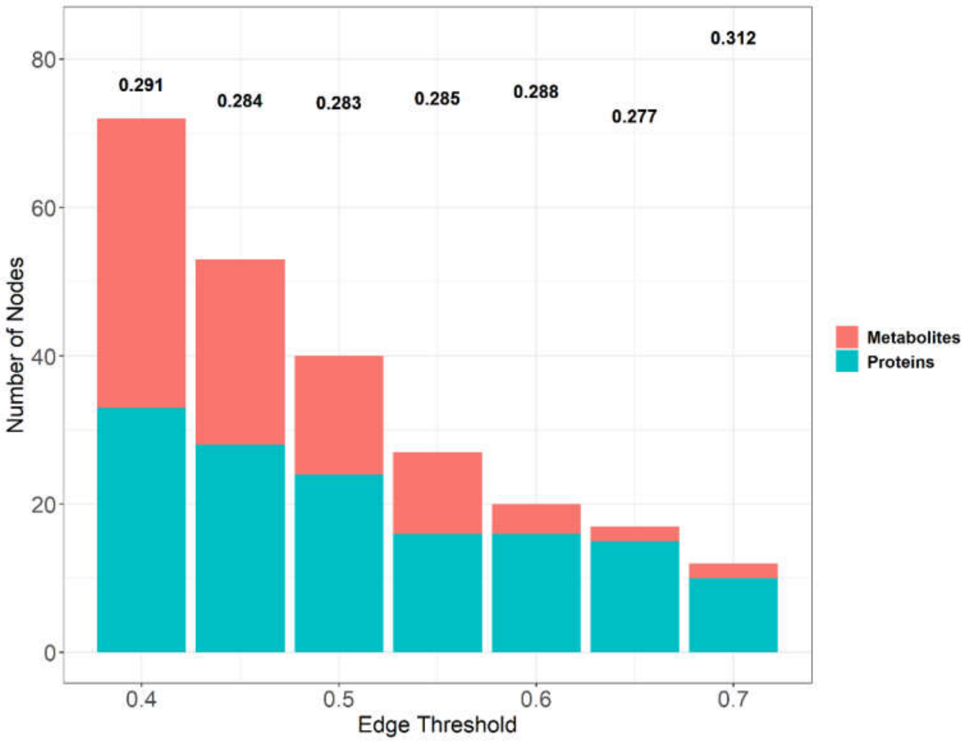

Figure S6: Effect of varying the edge threshold on the FEV$_1$% network constructed on unadjusted -omic data. Bottom axis displays the values for edge thresholds. Module 2 did not have edges strong enough to pass an edge threshold. Number of proteins for each module are in blue and the number of metabolites for each module are in red. The correlation between the module's first principal component and FEV$_1$% is displayed as the bolded value on each bar plot. The height of the correlation corresponds with the absolute value of the correlation.

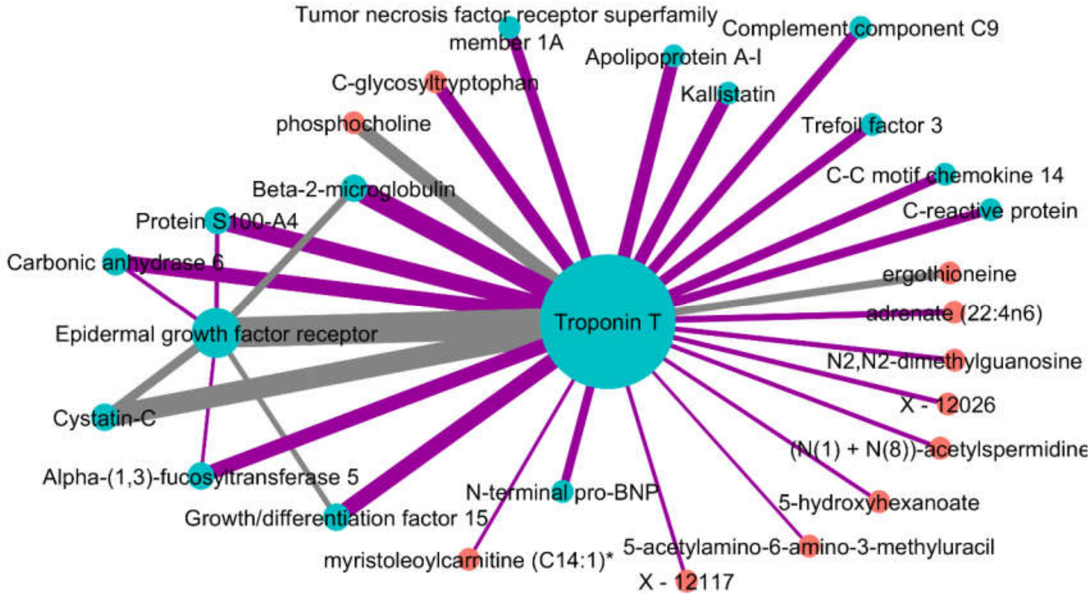

Figure S7: Identified network after applying SmCCNet to unadjusted proteomic data, metabolomic data and FEV_1%. Proteins are blue nodes and metabolites are red nodes. Grey edges represent a negative correlation between the nodes. Purple edges represent a positive correlation between the nodes. Edge thickness corresponds to the relationships between the nodes based on canonical weights.

Table S3: Individual Network Node Correlations to FEV$_1$%

		Correlation to FEV$_1$%
**Proteins**	Troponin T	-0.313
	Protein S100-A4	0.243
	Carbonic anhydrase 6	0.224
	Alpha-(1,3)-fucosyltransferase 5	-0.222
	Epidermal growth factor receptor	0.218
	C-reactive protein	-0.209
	Kallistatin	0.204
	Complement component C9	-0.201
	Apolipoprotein A-I	0.187
	N-terminal pro-BNP	-0.157
	Growth/differentiation factor 15	-0.156
	C-C motif chemokine 14	-0.142
	Trefoil factor 3	-0.139
	Cystatin-C	-0.138
	Beta-2-microglobulin	-0.123
	Tumor necrosis factor receptor superfamily member 1A	-0.115
**Metabolites**	phosphocholine	0.248
	ergothioneine	0.220
	5-hydroxyhexanoate	-0.211
	myristoleoylcarnitine (C14:1)	-0.198
	(N(1) + N(8))-acetylspermidine	-0.188
	X – 12026	-0.180
	C-glycosyltryptophan	-0.178
	adrenate (22:4n6)	-0.177
	N2,N2-dimethylguanosine	-0.163
	X – 12117	-0.150
	5-acetylamino-6-amino-3-methyluracil	-0.148

Pearson correlations between FEV$_1$% and individual metabolites and proteins in identified network associated with FEV$_1$%.

S.7. Network Comparison between Adjusted and Unadjusted -Omic Data with FEV$_1$%

When comparing the FEV$_1$% protein-metabolite network constructed on the adjusted -omic data with the network from the unadjusted -omic data, there was a significant number of protein and metabolite nodes consistent between the two networks (Fisher's exact test p-value = 8.2×10^{-20}) (Table S4).

Table S4. FEV$_1$% Network Nodes using Adjusted and Unadjusted Data.

Nodes in Adjusted Network	Nodes in Both Networks	Nodes in Unadjusted Network

RGMA*<br>Hemojuvelin<br>Macrophage mannose receptor 1<br>Angiopoietin-2<br>RBP<br>*Palmitoleoylcarnitine(C16:1)**<br>*Myristoleylcarnitine(C14:1)**<br>*Cis-4-decenoylcarnitine(C10:1)**	Troponin T* †<br>Protein S100-A4*†<br>Carbonic anhydrase 6*†<br>Alpha-(1,3)-fucosytransferase 5*†<br>Epidermal growth factor receptor*†<br>C-reactive protein†<br>Kallistatin†<br>Complement component C9 †<br>*Phosphocholine**†<br>*Ergothioneine**†<br>*5-hydroxyhexanoate**†<br>*(N (1) + N (8))- acetylspermidine**†	Apolipoprotein A-I †<br>N-terminal pro-BNP †<br>Growth/differentiation factor 15 †<br>C-C motif chemokine 14<br>Trefoil factor 3<br>Cystatin C<br>Beta-2-microglobulin<br>Tumor necrosis factor receptor super family member 1A<br>*Myristoleoylcarnitine (C14:1)* †<br>*C-glycosyltryptophan* †<br>*Adrenate (22:4n6)* †<br>*N2, N2-dimethylguanosine* †<br>*5-acetylamino-6-amino-3-methyluracil*<br>*X-12026* †<br>*X-12117*

Protein and metabolite nodes (italicized) in identified FEV_1% networks when SmCCNet was applied to adjusted and unadjusted -omic data. Middle, shaded column lists nodes in both networks. There was a significant number of protein and metabolite nodes consistent between the two networks (Fisher's exact test p-value = 8.2×10^{-20}). Proteins and metabolites (adjusted for blood cell count) that have greater than a 0.15 correlation to FEV_1% (FDR adjusted p-value < 0.001) are denoted with *. Unadjusted proteins and metabolites that have greater than a 0.15 correlation to FEV_1% (FDR adjusted p-value < 0.001) are denoted with †.

S.8. Parameter Selection for Percent Emphysema Modules using Adjusted -Omic Datasets

Similar to methods used to construct protein-metabolite networks correlated for FEV_1%, we tried different scaling constants for b and c where $b = c$ when applying scaled SmCCNet to adjusted proteomic and metabolomic data and percent emphysema. First, scaling constant values of 5, 10, 15, and 20 were applied to SmCCNet. Next, further analysis of scaling constant values between 10 and 20 was performed to determine the scaling constant for which module results ceased to have substantial changes in the diagnostics mentioned above. After analyzing the results from each scaling constant change, a final value was selected based on each module's first principal component correlation to percent emphysema, number of metabolites and proteins and strength of module edges for each module.

While all scaling constant changes resulted in a similar maximum module correlation to percent emphysema (rho = -0.29), after the scaling constant value of 15, the number of proteins and metabolites do not change much (Table S5). Scaling constant value of 15 was selected because it had the same correlation to percent emphysema as other modules from different scaling constants. However, it was the largest scaling constant that provided a large number of nodes for which to build a network before a sharp decrease in number of nodes. The largest module (Module 1) derived from SmCCNet with scaling constant of 15 is comprised of 258 nodes, while scaling constant of 16 gives a single module with 17 nodes.

Finalizing the use of scaling constant 15 and applying scaled SmCCNet to adjusted proteomic and metabolomic data resulted in three protein-metabolite modules correlated with percent emphysema. The optimal penalty parameters were (0.35, 0.15) which favors networks with more metabolites and fewer proteins. Not only did we examine the full module's correlation to percent emphysema, but it was important to inspect the individual correlations of proteins and metabolites in each module to percent emphysema (Figure S8). All proteins and metabolites not included in Module 1, 2 or 3 are represented as Module 0. Module 0 has a lower correlation with percent emphysema (rho = -0.068, p-value = 0.038) compared to Module 1 (rho = -0.29, p-value = 5.6×10^{-22}, 42 proteins, 216 metabolites), Module 2 (rho = -0.13, p-value = 0.00011, two proteins, one metabolite), or Module 3 (rho = -0.18, p-value = 3×10^{-8}, 23 proteins, one metabolite) correlation to percent emphysema.

Table S5: Different Scaling Constants for SmCCNet Applied to Percent Emphysema and Adjusted -Omic Data

Scaling Constant (*a, b, c*)	Network Module	Correlation (p-value)	Number of Proteins	Number of Metabolites	Total Nodes
(1, 10, 10)	1	-0.29 (3.0×10^{-19})	39	232	271
	2	-0.14 (1.5×10^{-5})	4	2	6
(1, 13, 13)	1	-0.29 (6.6×10^{-20})	37	216	253

	2	-0.13 (5.4 × 10^{-5})	2	2	4
(1, 15, 15)	1	-0.29 (5.6 × 10^{-20})	42	216	258
	2	-0.13 (0.00011)	2	1	3
	3	-0.18 (3.0 × 10^{-8})	23	1	24
(1, 16, 16)	1	-0.29 (3.6 × 10^{-20})	11	6	17
(1, 17, 17)	1	-0.29 (3.6 × 10^{-20})	11	6	17
(1, 20, 20)	1	-0.27 (5.1 × 10^{-17})	4	7	11

For each scaling weight change, the module diagnostics are reported. Correlation refers to the module's first principal component correlation to percent emphysema.

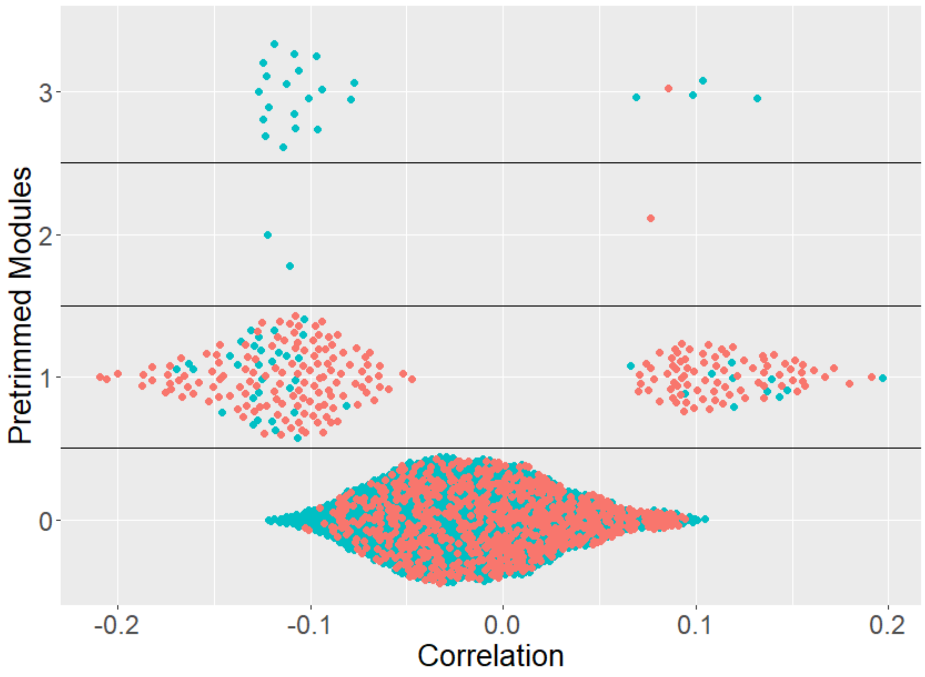

Figure S8. Correlations with percent emphysema for full module. All proteins and metabolites not included in Module 1, 2 or 3 are represented as Module 0. The proteins are blue and the metabolites are red. Module 0 has a -0.068 (p-value = 0.038) correlation, Module 1 has a -0.29 (p-value = 5.6 × 10^{-20}) correlation, Module 2 has a -0.13 (p-value = 0.00011) correlation, and Module 3 has a -0.18 (p-value = 3 × 10^{-8}) correlation to percent emphysema.

S.9. Identified Network Associated with Percent Emphysema

While the initial Module 1 provides a candidate network, it is very large (42 proteins, 216 metabolites) and may contain weak edges between features. To focus on the strongest connections within the network, edges that do not meet a specified edge threshold were removed from the network thereby removing metabolites and proteins that are weakly connected to other proteins and metabolites. Edge thresholds between 0.3 and 0.6 were evaluated. The resulting networks were summarized by the network's correlation to percent emphysema and the number of proteins and metabolites (Figure S9). Module 1 was the only module that passed edge cuts. Edge threshold value of 0.5 was chosen as the final edge threshold because it resulted in an interpretable network with 23 nodes. It also had the strongest edges and a balanced metabolite to protein ratio while still yielding a high correlation to percent emphysema (rho = -0.27, p-value = 2.6×10^{-17}).

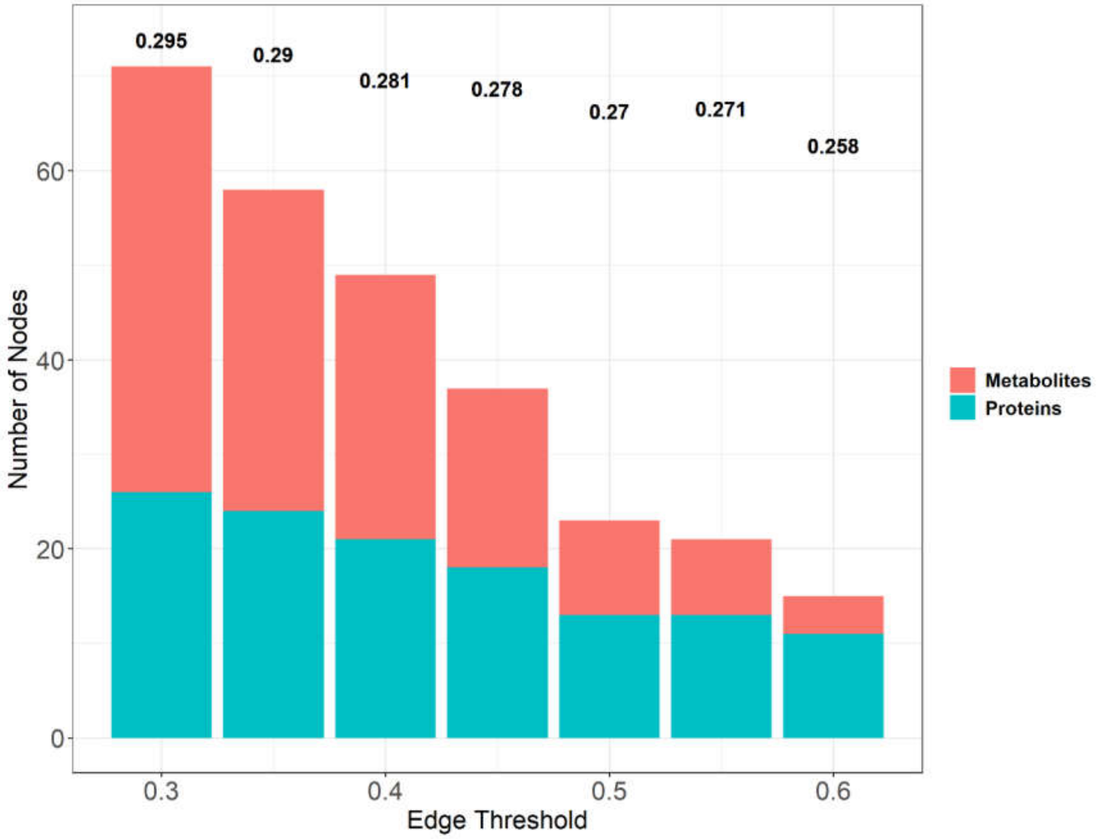

Figure S9: Effect of varying the edge threshold on the percent emphysema network constructed on adjusted -omic data. Bottom axis displays the values for edge thresholds. Module 2 and Module 3 did not have edges strong enough to pass an edge threshold. Number of proteins for each module are in blue and the number of metabolites for each module are in red. The correlation between the module first principal component and percent emphysema is displayed as the bolded value on each bar plot. The height of the correlation corresponds with the absolute value of the correlation.

S.10 Percent Emphysema Modules (Unadjusted -Omic Data)

Similar to methods used to construct previous protein-metabolite networks, we tried different scaling constants for *b* and *c* when applying scaled SmCCNet to unadjusted -omic data sets and percent emphysema (see Methods and the main manuscript about diagnostics for selecting constants). First, scaling constant values of 5, 10, 15, and 20 were applied to SmCCNet. Next, further

analysis of scaling constant values between 2 and 10 was performed to determine the scaling constant for which module results ceased to have substantial changes in previously mentioned diagnostics.

After scaling constant value of 7, the number of proteins and metabolites and the module correlation to percent emphysema did not change much (Table S6). Scaling constant value of 7 was selected as the final scaling constant for SmCCNet applied to percent emphysema and unadjusted proteomic and metabolomic data. Although the resulting Module 1 has a small number of proteins compared to number of metabolites (six proteins, 193 metabolites), the correlation to percent emphysema is high (rho = -0.30, p-value = 8.6 × 10^{-22}). Scaling constant values between 2 and 6 did yield modules with high correlations to percent emphysema and a more equal ratio between number of proteins and number of metabolites (e.g., scaling constant = 5, module 2). However, after further investigation, the modules' edges were weak and did not pass a high enough edge threshold.

Finalizing the use of scaling constant 7 and applying SmCCNet to unadjusted proteomic and metabolomic data and percent emphysema resulted in six modules. The optimal penalty parameters were (0.35, 0.55) which favors protein-metabolite networks with more metabolites and less proteins. In addition to the module's first principal component correlation to percent emphysema, the individual correlations of module metabolites and proteins to percent emphysema were also examined (Figure S10). Module 0 has a much lower correlation (rho = -0.08, p-value = 0.011) with percent emphysema compared to Module 1 (rho = -0.26, p-value = 8.6 × $10^{-22,}$ six proteins, 193 metabolites) correlation, Module 2 (rho = 0.20, p-value = 1.4 × 10^{-10}, three proteins, fourteen metabolites) correlation, Module 3 (rho = -0.13, p-value = 2.1 × 10^{-05}, one protein, 30 metabolites) correlation, Module 4 (rho = -0.20, p-value = 5.1 × 10^{-10}, one protein, eleven metabolites) correlation, Module 5 (rho = -0.11, p-value = 9.3 × 10^{-4}, two proteins, eleven metabolites) correlation, and Module 6 (rho = 0.16, p-value = 2.3 × 10^{-5}, one protein, one metabolite) to percent emphysema.

S.11. Identified Network associated with Percent Emphysema (Unadjusted -Omic Data)

While Module 1 is a candidate network, it contains weak edges and features only connected by weak edges. To focus on the strongest edges, we evaluated different edge thresholds values between 0.2 and 0.45. Resulting networks were summarized by network correlation to percent emphysema and number of proteins and metabolites (Figure S11). Module 1 was the only network that passed edge cuts. Edge threshold value of 0.4 was chosen as the final edge threshold because it resulted in a network with an interpretable number of 24 nodes while maintaining a high correlation to percent emphysema (rho = -0.26, p-value = 3.9 × 10^{-16}). The identified network has three proteins and 21 metabolites with a range of individual feature correlations to percent emphysema (Figure S12, Table S7). Although there are a low number of proteins in this network, the proteins are connected by highly weighted edges and have high connectivity. Growth hormone receptor, a protein, is the largest network hub with every other metabolite and protein connected to growth hormone receptor. The two most heavily weighted edges connect growth hormone receptor to apolipoprotein E and to proto-oncogene tyrosine-protein kinase receptor, both of which are proteins.

Table S6: Different Scaling Constants for SmCCNet Applied to Percent Emphysema and Unadjusted -Omic Data

Scaling Constant (*a, b, c*)	Network Module	Correlation (p-value)	Number of Proteins	Number of Metabolites	Total Nodes
	1	0.26 (7.5 × 10^{-16})	27	63	90
	2	-0.06 (0.048)	36	34	70
	3	-0.22 (1.3 × 10^{-11})	10	29	39
(1, 2, 2)	4	-0.09 (0.0055)	12	20	32
	5	-0.04 (0.23)	140	217	357
	6	-0.11 (0.00097)	5	9	14
	7	-0.15 (5.3 × 10^{-6})	2	5	7

	8	-0.13 (5.9 × 10^{-5})	3	1	4
(1, 3, 3)	1	-0.06 (0.051)	127	326	453
	2	0.26 (1.9 × 10^{-16})	47	178	225
	3	-0.08 (0.014)	18	13	31
	4	-0.13 (3.3 × 10^{-5})	2	3	5
	5	-0.14 (1.7 × 10^{-5})	19	1	20
	6	-0.10 (0.0017)	10	2	12
	7	0.05 (0.099)	1	1	2
(1, 4, 4)	1	0.05 (0.16)	280	88	368
	2	-0.10 (0.0013)	218	112	330
	3	-0.20 (6.5 × 10^{-10})	7	8	15
	4	-0.12 (0.00034)	64	20	84
	5	0.13 (4.4 × 10^{-5})	2	3	5
(1, 5, 5)	1	0.08 (0.0096)	141	75	216
	2	-0.24 (1.4 × 10^{-13})	10	10	20
	3	-0.11 (0.00048)	116	124	240
	4	-0.02 (0.5)	10	29	39
	5	-0.18 (1.4 × 10^{-8})	2	6	8
	6	-0.11 (0.00059)	4	1	5
	7	-0.12 (0.00019)	22	15	37
	8	-0.16 (4.8 × 10^{-7})	2	6	8
	9	-0.09 (0.0081)	2	3	5
(1, 6, 6)	1	0.05 (0.11)	374	84	458
	2	-0.20 (6.6 × 10^{-10})	13	25	38
	3	-0.12 (0.00026)	382	116	498
	4	-0.20 (7.1 × 10^{-10})	2	6	8
	5	-0.12 (0.00018)	19	11	30
	6	-0.11 (0.00048)	19	13	32
	7	-0.10 (0.0015)	22	4	26
	8	-0.03 (0.3)	12	2	14
	9	-0.07 (0.035)	2	3	5
	10	0.03 (0.28)	5	1	6
(1, 7, 7)	1	-0.30 (8.6 × 10^{-22})	6	193	199
	2	0.21 (1.4 × 10^{-10})	3	14	17
	3	-0.14 (2.1 × 10^{-10})	1	30	31
	4	-0.20 (5.1 × 10^{-10})	1	11	12
	5	-0.11 (0.00093)	2	11	13
	6	-0.14 (2.3 × 10^{-5})	1	1	2
(1, 10, 10)	1	-0.31 (3.8 × 10^{-22})	6	203	209
	2	0.15 (5.0 × 10^{-6})	2	5	7
	3	-0.14 (7.8 × 10^{-6})	1	1	2
	4	-0.21 (3.6 × 10^{-11})	3	10	13
	5	0.13 (6.3 × 10^{-5})	1	1	2
(1, 13, 13)	1	-0.31 (1.7 × 10^{-22})	8	219	227
	2	-0.26 (1.1 × 10^{-16})	4	3	7
	3	-0.15 (3.4 × 10^{-6})	1	2	3
(1, 15, 15)	1	-0.31 (5.5 × 10^{-23})	4	216	220
	2	-0.25 (4.1 × 10^{-15})	9	14	23
	3	-0.15 (1.9 × 10^{-6})	1	1	2
(1, 20, 20)	1	-0.31 (4.3 × 10^{-23})	9	225	234
	2	-0.25 (2.0 × 10^{-14})	5	2	7

For each scaling weight change, the module diagnostics are reported. Correlation refers to the module's first principal component correlation to percent emphysema.

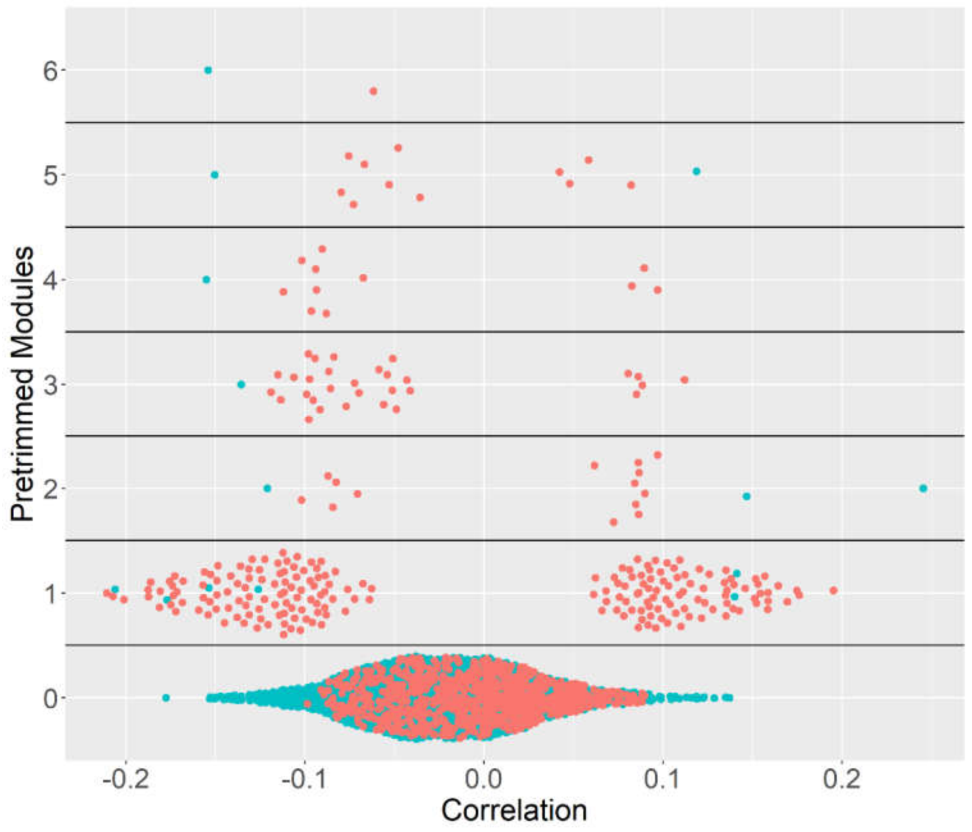

Figure S10: Correlations with percent emphysema for full module. All proteins and metabolites not included in modules 1 through 6 are represented as Module 0. The proteins are blue and the metabolites are red.

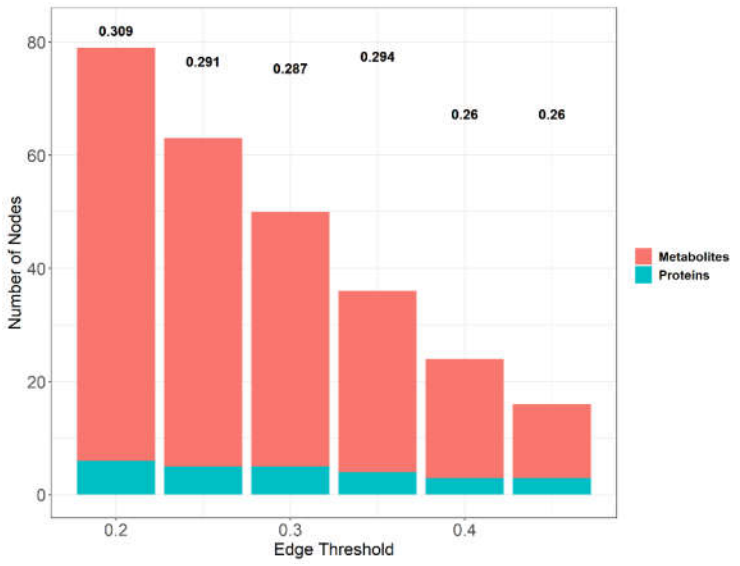

Figure S11: Effect of varying the edge threshold on the percent emphysema network constructed on unadjusted -omic data. Bottom axis displays the values for edge thresholds. Number of proteins for each module are in blue and the number of metabolites for each module are in red. The correlation between the module first principal component and percent emphysema is displayed as the bolded value on each bar plot. The height of the correlation corresponds to the absolute value of the correlation.

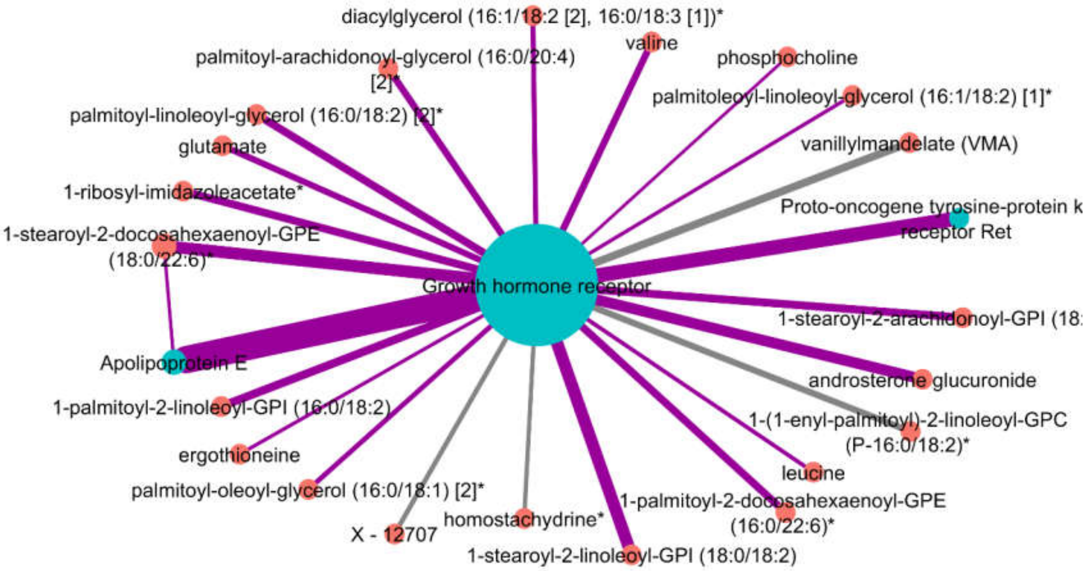

Figure S12: Identified network after applying SmCCNet to unadjusted proteomic data, metabolomic data and percent emphysema. Proteins are blue nodes and metabolites are red nodes. Grey edges represent a negative correlation between the nodes. Purple edges represent a positive correlation between the nodes. Edge thickness corresponds to the product of the canonical weights of the two nodes. Node size corresponds with connectivity.

Table S7: Individual Network Node Correlations to Percent Emphysema

		**Correlation to Percent Emphysema**
**Proteins**	Growth hormone receptor	-0.206
	Proto-oncogene tyrosine-protein kinase receptor Ret	-0.177
	Apolipoprotein E	-0.154
**Metabolites**	androsterone glucuronide	-0.211
	1-stearoyl-2-linoleoyl-GPI (18:0/18:2)	-0.207
	1-stearoyl-2-docosahexaenoyl-GPE (18:0/22:6)	-0.201
	vanillylmandelate (VMA)	0.195
	1-palmitoyl-2-docosahexaenoyl-GPE (16:0/22:6)	-0.188
	Ergothioneine	-0.187
	1-palmitoyl-2-linoleoyl-GPI (16:0/18:2)	-0.186
	Phosphocholine	-0.181
	Homostachydrine	0.176
	1-ribosyl-imidazoleacetate*	-0.173
	Valine	-0.172
	palmitoyl-linoleoyl-glycerol (16:0/18:2) [2]	-0.168
	X – 12707	0.164
	1-stearoyl-2-arachidonoyl-GPI (18:0/20:4)	-0.159
	Leucine	-0.157
	Glutamate	-0.156

palmitoyl-arachidonoyl-glycerol (16:0/20:4) [2]	-0.153
palmitoleoyl-linoleoyl-glycerol (16:1/18:2) [1]	-0.141
diacylglycerol (16:1/18:2 [2], 16:0/18:3 [1])	-0.138
palmitoyl-oleoyl-glycerol (16:0/18:1) [2]	-0.138
1-(1-enyl-palmitoyl)-2-linoleoyl-GPC (P-16:0/18:2)	0.135

Pearson correlations between percent emphysema and individual metabolites and proteins in identified network associated with percent emphysema.

S.12. Network Comparison between Adjusted and Unadjusted -Omic Data with Percent Emphysema

When comparing the percent emphysema protein-metabolite network constructed on the adjusted -omic data with the network from the unadjusted -omic data, there was a significant number of protein and metabolite nodes consistent between the two networks (Fisher's exact test p-value = 7.8×10^{-20}) (Table S8).

Table S8. Nodes of Percent Emphysema Networks using Adjusted and Unadjusted Data.

Nodes in Adjusted Network	Nodes in Both Networks	Nodes in Unadjusted Network
Troponin T*	Growth hormone receptor*†	*Vanillylmandelate (VMA) †*
Leptin*	Proto-onogene tyrosine-protein kinase receptor Ret†	*Ergotioneine†*
Glucagon*	Apolipoprotein E†	*Phosphocholine†*
Chordin-like protein 1	*1-stearoyl-2-linoleoyl-GPI (18:0/18:2)* †*	*Homostachydrine†*
Hemojuvelin	*Androsterone glucuronide*†*	*1-ribosyl-imidazoleacetate†*
Sex hormone-binding globulin	*1-stearoyl-2-docosahexaenoyl-GPE (18:0/22:6)* †*	*Leucine†*
Aminoacylase-1	*1-palmitoyl-2-docosahexaenoyl-GPE (18:0/22:6)* †*	*Palmitoyl-arachidonoyl-glycerol (16:0/20:4) [2] †*
Adiponectin	*1-palmitoyl-2-linoleoyl-GPI (16:0/18:2)* †*	*Palmitoleoyl-lineoleoyl-glycerol (16:1/18:2) [1]*
IGFBP-2	*Valine* †*	*Diacylglycerol (16:1/18:2 [2], 16:0/18:3 [1])*
QORL1	*Palmitoyl-linoleoyl-glycerol (16:0/18:2) [2]* †*	*Palmitoyl-oleoyl-glycerol (16:0/18:1) [2]*
*1-ribosyl-imidazoleacetate**	*1-stearoyl-2-arachidonoyl-GPI (18:0/20:4)* †*	*1-(1-enyl-palmitoyl)-2-linoleoyl-GPC (P-16:0/18:2)*
	Glutamate †*	*X-12707†*

Protein nodes and metabolite nodes (italicized) in identified percent emphysema networks when SmCCNet was applied to adjusted and unadjusted -omic data. Middle, shaded column lists nodes in both networks. There is a significant number of protein and metabolite nodes consistent between the two networks (Fisher's exact test p-value = 7.8×10^{-20}). Proteins and metabolites (adjusted for blood cell count) that have greater than a 0.15 correlation to percent emphysema (FDR adjusted p-value < 0.001) are denoted with *. Unadjusted proteins and metabolites that have greater than a 0.15 correlation to percent emphysema (FDR adjusted p-value < 0.001) are denoted with †.

S.13. Secondary Network Analysis

Secondary analysis was calculated on the subjects to look at trends with each network constructed on adjusted -omic data by different clinical subsets (Figure S13, Figure S14). There was a significant difference (p-value < 0.0001) between subjects who had zero or at least one exacerbation for the FEV_1% network, but there was no significant difference for the percent emphysema network (Figure S13). There was a significant difference (p-value < 0.0001) between subjects with and without cardiovascular comorbidity for the FEV_1% network but not within the network associated with percent emphysema (Figure S14).

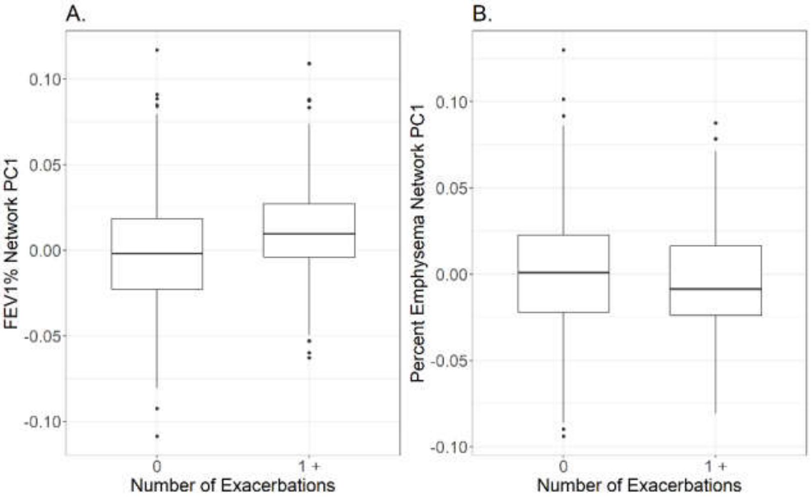

Figure S13. Association of network first principal component (PC1) with exacerbations. Subjects were separated by those who had zero and those who has at least one exacerbation and evaluated for trends within the A) FEV_1% or B) percent emphysema networks.

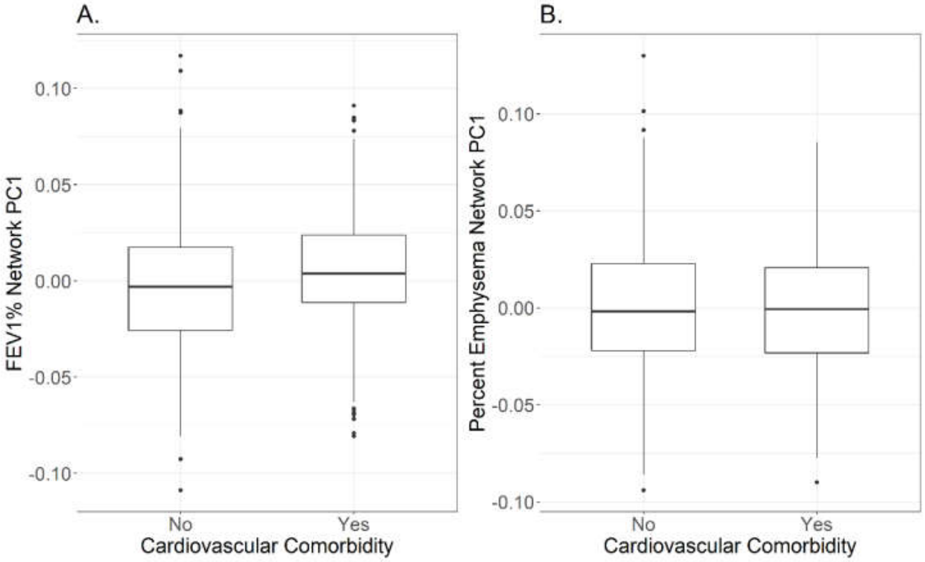

Figure S14. Association of network PC1 with cardiovascular comorbidity. Subjects were separated by those with or without cardiovascular comorbidities and evaluated for trends within the A) FEV_1% or B) percent emphysema networks.

S.14. Methods Supplement

Protein-metabolite networks correlated to FEV_1% and percent emphysema were constructed using SmCCNet (Figure S15), a technique developed by Shi et al [15] that uses multiple canonical correlation network analysis to integrate multiple -omics data types with a phenotype of interest. The objective function is $(w_1, w_2) = max\ [\ aw_1 X_1^T X_2 w_2 + bw_1 X_1 Y + cw_2 X_2 Y]$ subject to $||w_s||^2 = 1$, $P_s(w_s) \leq c_s$, $s = 1, 2$ where X_1 and X_2 are -omic data sets (metabolites and proteins respectively), Y is a phenotype of interest (FEV_1% or percent emphysema), w_1 and w_2 are canonical weights, a, b, and c are scaling constants, and c_1, c_2 are penalty parameters under the l_1 – norm function. The sparse penalty parameters were chosen through a 5-fold cross validation (Figure S15, Step 1) to find the penalty pair that minimized prediction error. All penalty pairs from the set (0.05, 0.15, 0.25, 0.35, 0.45, 0.55) were tested in a grid search to find the optimal (c_1, c_2). The c_1 penalty is imposed on the metabolic data while the c_2 penalty is imposed on the proteomic data. Small penalties correspond with less of that particular -omic type contributing to the network. Conversely, a high penalty parameter should result in more of that -omic type included in the network. Canonical weights indicate the contribution of metabolites and proteins to the canonical correlations and were generated through sparse multiple canonical correlation analysis (Figure S15, Step 2).

An important step in SmCCNet is the feature subsampling step to create robust network construction. The proteomic and metabolomic data was subsampled 500 times and a relationship matrix was created for each subsample based on the canonical weights. The proportions of subsampled protein and metabolite features were 70% for both feature sets. To find the similarity matrix, all 500 relationship matrices were averaged and rescaled to have a maximum relatedness of one. A hierarchical tree was constructed based on the averaged and rescaled relationship matrix (Figure S15, Step 3). A height threshold of one was applied to the tree, resulting in protein-metabolite modules correlated to the phenotype of interest. A more complete description of SmCCNet can be found in Shi et al 2019 [15].

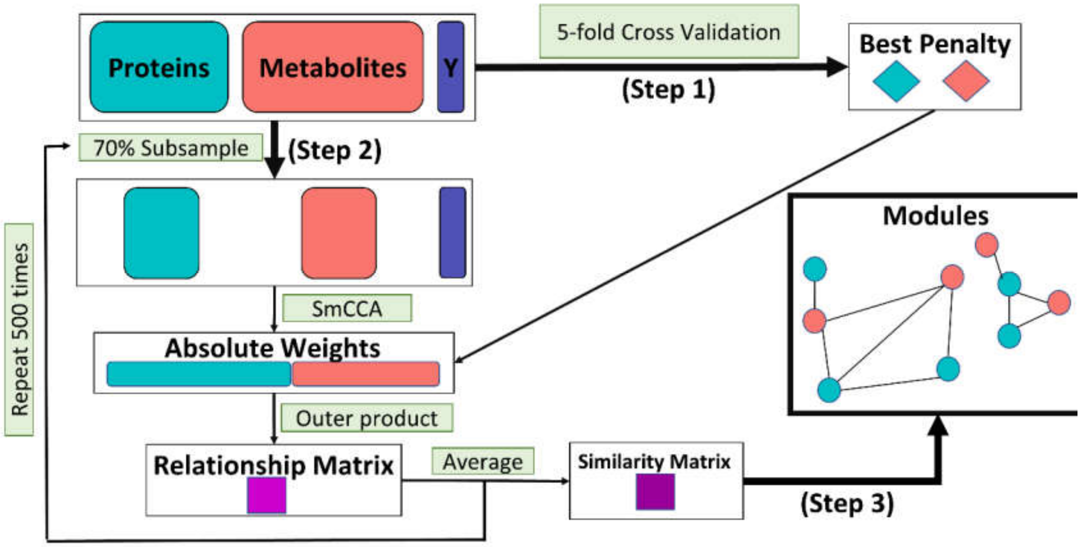

Figure S15: SmCCNet workflow [15]. Step 1: Find the best sparse penalty parameters through a 5-Fold cross validation. Step 2: Subsample the proteomic and metabolomic data, find the canonical weights using the penalty pair found in step 1, create a relationship matrix, repeat 500 times. Compute similarity matrix by

averaging and scaling all 500 relationship matrices. Step 3: Find protein-metabolite modules by applying a hierarchical tree cut to similarity matrix.

S.15. Metabolon and Data Processing

Samples were extracted with methanol under vigorous shaking for two minutes (Glen Mills GenoGrinder 2000) followed by centrifugation to remove protein, dissociate small molecules bound to protein or trapped in the precipitated protein matrix, and to recover chemically diverse metabolites. The resulting extract is divided into five fractions: two for analysis by two separate reverse phase (RP)/UPLC-MS/MS methods with positive ion mode electrospray ionization (ESI), one for analysis by RP/UPLC-MS/MS with negative ion mode ESI, one for analysis by HILIC/UPLC-MS/MS with negative ion mode ESI, and one sample is reserved for backup. Metabolon has developed peak detection and integration software to generate a list of *m*/*z* ratios, retention indices and area under the curve (AUC) values for each detected metabolite, as described in detail [49-51]. User specified criteria for peak detection included thresholds for signal to noise ratio, area and width. Relative standard deviations (RSDs) of peak area were determined for internal and recovery standards to confirm extraction efficiency, instrument performance, column integrity, chromatography and mass calibration. The biological data sets, including QC samples, were chromatographically aligned based on a retention index that utilized internal standards assigned a fixed RI value. The RI of the experimental peak was determined by assuming a linear fit between flanking RI markers whose RI values are set. Peaks were matched against an in-house library of authentic standards and routinely detected unknown compounds specific to the respective method. Identifications were based on retention index values, experimental precursor mass match to the library authentic standard within 10 ppm, and quality of MS/MS match. All proposed identifications were then manually reviewed and curated by an analyst who approved or rejected each identification based on the criteria above. The platform reported 1,392 features, including 1,064 annotated features which were grouped by Metabolon into "Super Pathways" including: 436 lipids, 261 xenobiotics, 207 amino acids, 40 peptides, 38 cofactors and enzymes, 35 nucleotides, 25 carbohydrates, 11 energy pathway compounds, and 11 partially characterized molecules. All compounds are further annotated by "Sub Pathway" (e.g. "sphingomyelins", "carnitine metabolism", "lysine metabolism").

S.16. Acknowledgements Supplement

COPD Foundation Funding

The COPDGene® project is also supported by the COPD Foundation through contributions made to an Industry Advisory Board comprised of AstraZeneca, Boehringer-Ingelheim, Genentech, GlaxoSmithKline, Novartis, and Sunovion.

COPDGene® Investigators – Core Units

Administrative Center: James D. Crapo, MD (PI); Edwin K. Silverman, MD, PhD (PI); Barry J. Make, MD; Elizabeth A. Regan, MD, PhD

Genetic Analysis Center: Terri Beaty, PhD; Ferdouse Begum, PhD; Peter J. Castaldi, MD, MSc; Michael Cho, MD; Dawn L. DeMeo, MD, MPH; Adel R. Boueiz, MD; Marilyn G. Foreman, MD, MS; Eitan Halper-Stromberg; Lystra P. Hayden, MD, MMSc; Craig P. Hersh, MD, MPH; Jacqueline Hetmanski, MS, MPH; Brian D. Hobbs, MD; John E. Hokanson, MPH, PhD; Nan Laird, PhD; Christoph Lange, PhD; Sharon M. Lutz, PhD; Merry-Lynn McDonald, PhD; Margaret M. Parker, PhD; Dmitry Prokopenko, Ph.D; Dandi Qiao, PhD; Elizabeth A. Regan, MD, PhD; Phuwanat Sakornsakolpat, MD; Edwin K. Silverman, MD, PhD; Emily S. Wan, MD; Sungho Won, PhD

Imaging Center: Juan Pablo Centeno; Jean-Paul Charbonnier, PhD; Harvey O. Coxson, PhD; Craig J. Galban, PhD; MeiLan K. Han, MD, MS; Eric A. Hoffman, Stephen Humphries, PhD; Francine L. Jacobson, MD, MPH; Philip F. Judy, PhD; Ella A. Kazerooni, MD; Alex Kluiber; David A. Lynch,

MB; Pietro Nardelli, PhD; John D. Newell, Jr., MD; Aleena Notary; Andrea Oh, MD; Elizabeth A. Regan, MD, PhD; James C. Ross, PhD; Raul San Jose Estepar, PhD; Joyce Schroeder, MD; Jered Sieren; Berend C. Stoel, PhD; Juerg Tschirren, PhD; Edwin Van Beek, MD, PhD; Bram van Ginneken, PhD; Eva van Rikxoort, PhD; Gonzalo Vegas Sanchez-Ferrero, PhD; Lucas Veitel; George R. Washko, MD; Carla G. Wilson, MS;

PFT QA Center, Salt Lake City, UT: Robert Jensen, PhD

Data Coordinating Center and Biostatistics, National Jewish Health, Denver, CO: Douglas Everett, PhD; Jim Crooks, PhD; Katherine Pratte, PhD; Matt Strand, PhD; Carla G. Wilson, MS

Epidemiology Core, University of Colorado Anschutz Medical Campus, Aurora, CO: John E. Hokanson, MPH, PhD; Gregory Kinney, MPH, PhD; Sharon M. Lutz, PhD; Kendra A. Young, PhD

Mortality Adjudication Core: Surya P. Bhatt, MD; Jessica Bon, MD; Alejandro A. Diaz, MD, MPH; MeiLan K. Han, MD, MS; Barry Make, MD; Susan Murray, ScD; Elizabeth Regan, MD; Xavier Soler, MD; Carla G. Wilson, MS

Biomarker Core: Russell P. Bowler, MD, PhD; Katerina Kechris, PhD; Farnoush Banaei-Kashani, Ph.D

COPDGene® Investigators – Clinical Centers

Ann Arbor VA: Jeffrey L. Curtis, MD; Perry G. Pernicano, MD

Baylor College of Medicine, Houston, TX: Nicola Hanania, MD, MS; Mustafa Atik, MD; Aladin Boriek, PhD; Kalpatha Guntupalli, MD; Elizabeth Guy, MD; Amit Parulekar, MD;

Brigham and Women's Hospital, Boston, MA: Dawn L. DeMeo, MD, MPH; Alejandro A. Diaz, MD, MPH; Lystra P. Hayden, MD; Brian D. Hobbs, MD; Craig Hersh, MD, MPH; Francine L. Jacobson, MD, MPH; George Washko, MD

Columbia University, New York, NY: R. Graham Barr, MD, DrPH; John Austin, MD; Belinda D'Souza, MD; Byron Thomashow, MD

Duke University Medical Center, Durham, NC: Neil MacIntyre, Jr., MD; H. Page McAdams, MD; Lacey Washington, MD

Grady Memorial Hospital, Atlanta, GA: Eric Flenaugh, MD; Silanth Terpenning, MD

HealthPartners Research Institute, Minneapolis, MN: Charlene McEvoy, MD, MPH; Joseph Tashjian, MD

Johns Hopkins University, Baltimore, MD: Robert Wise, MD; Robert Brown, MD; Nadia N. Hansel, MD, MPH; Karen Horton, MD; Allison Lambert, MD, MHS; Nirupama Putcha, MD, MHS

Lundquist Institute for Biomedical Innovationat Harbor UCLA Medical Center, Torrance, CA: Richard Casaburi, PhD, MD; Alessandra Adami, PhD; Matthew Budoff, MD; Hans Fischer, MD; Janos Porszasz, MD, PhD; Harry Rossiter, PhD; William Stringer, MD

Michael E. DeBakey VAMC, Houston, TX: Amir Sharafkhaneh, MD, PhD; Charlie Lan, DO

Minneapolis VA: Christine Wendt, MD; Brian Bell, MD; Ken M. Kunisaki, MD, MS

National Jewish Health, Denver, CO: Russell Bowler, MD, PhD; David A. Lynch, MB

Reliant Medical Group, Worcester, MA: Richard Rosiello, MD; David Pace, MD

Temple University, Philadelphia, PA: Gerard Criner, MD; David Ciccolella, MD; Francis Cordova, MD; Chandra Dass, MD; Gilbert D'Alonzo, DO; Parag Desai, MD; Michael Jacobs, PharmD; Steven Kelsen, MD, PhD; Victor Kim, MD; A. James Mamary, MD; Nathaniel Marchetti, DO; Aditi Satti, MD; Kartik Shenoy, MD; Robert M. Steiner, MD; Alex Swift, MD; Irene Swift, MD; Maria Elena Vega-Sanchez, MD

University of Alabama, Birmingham, AL: Mark Dransfield, MD; William Bailey, MD; Surya P. Bhatt, MD; Anand Iyer, MD; Hrudaya Nath, MD; J. Michael Wells, MD

University of California, San Diego, CA: Douglas Conrad, MD; Xavier Soler, MD, PhD; Andrew Yen, MD

University of Iowa, Iowa City, IA: Alejandro P. Comellas, MD; Karin F. Hoth, PhD; John Newell, Jr., MD; Brad Thompson, MD

University of Michigan, Ann Arbor, MI: MeiLan K. Han, MD MS; Ella Kazerooni, MD MS; Wassim Labaki, MD MS; Craig Galban, PhD; Dharshan Vummidi, MD

University of Minnesota, Minneapolis, MN: Joanne Billings, MD; Abbie Begnaud, MD; Tadashi Allen, MD

University of Pittsburgh, Pittsburgh, PA: Frank Sciurba, MD; Jessica Bon, MD; Divay Chandra, MD, MSc; Carl Fuhrman, MD; Joel Weissfeld, MD, MPH

University of Texas Health, San Antonio, San Antonio, TX: Antonio Anzueto, MD; Sandra Adams, MD; Diego Maselli-Caceres, MD; Mario E. Ruiz, MD; Harjinder Singh
